# Foodborne Lactic Acid Bacteria Inactivate Planktonic and Sessile *Escherichia coli* O157:H7 in a Meat Processing Environment: A Physiological and Proteomic Study

**DOI:** 10.3390/foods14213670

**Published:** 2025-10-28

**Authors:** Lucia Cisneros, Ayelen Antonella Baillo, Diego Ploper, María Pia Valacco, Silvia Moreno, Osvaldo Yantorno, Vincenzina Fusco, Silvina Fadda

**Affiliations:** 1 Laboratory of Technology and Development: Meat and Meat Products (Tecno I), Reference Center for Lactobacilli—National Scientific and Technical Research Council—Miguel Lillo Foundation—Foundation for Education, Science and Culture (CERELA CONICET-FML-FCIC), Batalla de Chacabuco 145, San Miguel de Tucumán 4000, Tucumán, Argentina; luciacisneros86@gmail.com (L.C.); abaillo@cerela.org.ar (A.A.B.); 2 Institute for Research in Applied Molecular and Cellular Medicine (IMMCA)—National University of Tucumán—National Scientific and Technical Research Council—Provincial Health System (UNT-CONICET-SIPROSA), Pasaje Manuel Dorrego 1080, San Miguel de Tucumán 4000, Tucumán, Argentina; diegoploper@yahoo.com.ar; 3 Center for Chemical and Biological Studies by Mass Spectrometry (CEQUIBIEM) Department of Biological Chemistry, Faculty of Exact and Natural Sciences—University of Buenos Aires-Institute of Biological Chemistry of the Faculty of Exact and Natural Sciences—National Scientific and Technical Research Council (QB-FCEN UBA/IQUIBICEN-CONICET) Intendente Güiraldes 2160 Ciudad Universitaria, Capital Federal C1428EGA, Argentina; pvalacco@qb.fcen.uba.ar (M.P.V.); cequibiem-servicios@qb.fcen.uba.ar (S.M.); 4 Center for Research and Development in Industrial Fermentations—National Scientific and Technical Research Council (CINDEFI-CONICET), Faculty of Exact Sciences, National University of La Plata (UNLP), calle 50 e/115 y 116, La Plata 1900, Buenos Aires, Argentina; yantorno@quimica.unlp.edu.ar; 5 Institute of Sciences of Food Production, National Research Council of Italy, via Amendola 122/O, 70126 Bari, Italy

**Keywords:** lactic acid bacteria, EHEC, biofilm, bioprotection, meat, processing environment, proteomics

## Abstract

Enterohemorrhagic *Escherichia coli* (EHEC) forms persistent biofilms on meat processing surfaces, posing a significant cross-contamination risk. This study assessed the antagonistic capacity of lactic acid bacteria (LAB) against EHEC under meat-processing-like conditions. Three LAB strains were tested in planktonic co-culture with EHEC at 12 °C, all displaying bactericidal activity. In biofilm assays on stainless steel, LAB reduced EHEC biofilms without affecting their own viability. LAB cell-free supernatants further inhibited EHEC biofilms by 2.6–3.5 log CFU/cm^2^, highlighting the role of secreted antagonistic compounds. Among the tested strains, *Pediococcus pentosaceus* CRL 2145 showed the strongest effect and was selected for deeper analysis. Fluorescence microscopy confirmed EHEC cell death within mixed biofilms. Proteomic profiling of CRL 2145 under mixed-biofilm conditions revealed 162 differentially expressed proteins, with 156 upregulated. These proteins were mainly associated with metabolism, transcription, translation, and stress response pathways, suggesting a multifactorial inhibitory mechanism involving metabolic dominance, physical competition, and secretion of antagonistic molecules. Overall, this study deepens our understanding of the molecular and physiological aspects of LAB–EHEC interaction. *P. pentosaceus* CRL 2145 emerges as a promising biocontrol agent that could be applied, alone or with its supernatants, to meat processing surfaces to improve food safety. Proteomic data: ProteomeXchange PXD067300.

## 1. Introduction

In the food industry, surfaces that come into contact with food, as well as processing facilities in general, can be colonized by pathogenic microorganisms capable of forming biofilms. The persistence of these biofilms on abiotic surfaces is mainly driven by the enhanced tolerance of biofilm cells to disinfectants and their growth in hard-to-reach areas that are difficult to sanitize [1,2,3]. Thus, this is a matter of concern mainly for the food industry.

Nowadays, growing interest has been directed toward the development of biological approaches to control biofilms formed by pathogenic and spoilage microorganisms in food processing environments. As previously mentioned, this is largely due to the resistance and tolerance of biofilms to chemical disinfectants, as well as growing public awareness and preference for environmentally friendly alternatives. Conventional and novel methods to prevent and control *Escherichia coli* biofilm have been proposed [4]. Among the different biological approaches proposed for controlling biofilms produced by foodborne pathogens, attention has focused on the use of naturally derived compounds from bacteria or plants with GRAS (Generally Recognized As Safe) status, as well as on the application of bacteriophages [5,6]. Furthermore, bioprotective cultures offer the advantage of not causing undesirable sensory effects in food products, nor do they pose a risk of corrosion to metallic surfaces commonly used in food processing environments [7,8,9]. In this regard, the application of lactic acid bacteria (LAB) or their metabolites to inactivate enterohemorrhagic *Escherichia coli* (EHEC) and other pathogenic microorganisms has gained growing interest in recent years. For instance, Fretin et al. (2020) [10] evaluated the effectiveness of a bacterial consortium (*Hafnia alvei*, *Lactiplantibacillus plantarum*, and *Lactococcus lactis*) in reducing *E. coli* O26:H11 growth in raw pressed cheeses. Mahdhi et al. (2017) [11] studied the ability of exopolysaccharides produced by probiotic *L. plantarum* strains to inhibit biofilm formation by both Gram-positive and Gram-negative pathogens. Lastly, some LAB bacteriocins, which are typically active against Gram-positive bacteria, have been reported as active also against Gram-negative organisms [12,13,14]. Furthermore, LAB biofilms can exert a protective antagonistic role by hindering the attachment and subsequent biofilm development of pathogenic bacteria. Strategies most commonly evaluated for pathogen inhibition include competition for nutrients, alteration of the physico-chemical features of solid surfaces, and/or the production of antimicrobial compounds [15,16,17,18]. Recently, it was evidenced that foodborne LAB strains inhibited EHEC biofilms on inert surfaces at 12 °C via competition, exclusion, and displacement. Notably, *P. pentosaceus* CRL2145 showed the strongest competitive activity, as confirmed by physiological, proteomic, and electron microscopy analyses [19,20]. Although previous studies have demonstrated that foodborne LAB can inhibit EHEC biofilms, the molecular mechanisms underlying these interactions remain poorly understood. To fill this knowledge gap, the present study provides a comprehensive analysis of the antagonistic activity of selected LAB strains, with a particular emphasis on the mechanisms driving their interactions with *E. coli* NCTC 12900. The inhibitory activity of *P. pentosaceus* CRL 2145 and *Lactiplantibacillus plantarum* CRL 1482 and CRL 1075 was evaluated under planktonic conditions in a Meat Experimental System. In addition, LAB culture supernatants on stainless steel chips were tested to evidence inhibitory factors against EHEC biofilm. The persistence and behavior of LAB during sessile co-cultivation with EHEC were also assessed. Finally, a proteomic analysis of *P. pentosaceus* CRL 2145 during mixed-biofilm formation was performed, uncovering the metabolic basis of its competitive advantage. Altogether, this study moves beyond phenotypic observations by linking inhibition assays with molecular evidence, thereby providing novel mechanistic insights into LAB–EHEC interactions and establishing *P. pentosaceus* CRL2145 as a promising biocontrol agent for meat processing environments.

## 2. Materials and Methods

### 2.1. Bacterial Strains and Culture Conditions

The LAB strains used in this study were from the CERELA culture collection, namely *Pediococcus pentosaceus* CRL 2145, isolated from chickpea sourdough, *Lactiplantibacillus plantarum* CRL 1482, isolated from fermented sausage, and *L. plantarum* CRL 1075, isolated from peas. These strains are able to form biofilm and combat EHEC in vitro [19,20]. Long-term storage of these bacteria was carried out at −20 °C in milk yeast extract (10% *w*/*v* skim milk, 0.5% *w*/*v* yeast extract, and 1% glycerol), whereas working cultures were prepared in MRS (de Man, Rogosa, and Sharpe) broth (Britania, Buenos Aires, Argentina) at 30 °C for 16–18 h. *E. coli* O157:H7 NCTC 12900 (National Type Culture Collection, Colindale, London), which does not produce either enterotoxins Stx1 or Stx2 [21,22], was used as EHEC model. This strain has been shown to harbor several virulence- and biofilm-associated genes, thereby supporting its use as a representative O157:H7 model [20,23]. Long-term storage of this bacterium was carried out by addition of 20% glycerol and freezing at −80 °C in the Luria–Bertani (LB) broth culture. Working cultures of this strain were obtained from two transfers in LB broth at 37 °C.

### 2.2. Meat Experimental System (MES)

A Meat Experimental System (MES) containing soluble meat components was used as the growth medium for planktonic cultures and biofilm production assays [20,24]. To prepare the MES, bovine semimembranosus muscle was homogenized with deionized water (1:10 *w*/*v*) using a Stomacher 400 blender (Stomacher, London, UK). The extract was centrifuged (14,000× *g*, 20 min, 4 °C) and the supernatant filtered and sterilized (Steritop GP, Biopore, Buenos Aires, Argentina). The sterile extract was supplemented with 0.5% glucose, and its sterility was verified in Plate Count Agar (PCA). The MES was aliquoted and stored at −20 °C, and the same batch was used across all experiments to guarantee reproducibility.

### 2.3. Planktonic Growth Kinetics

Individual and co-cultures of both microorganisms were prepared to investigate their performances., In particular, each LAB strain was cultured individually in 100 mL Schott bottles and together with *E. coli* NCTC 12900 (LAB and EHEC were inoculated at 8 log CFU per mL in 70 mL of MES). The resulting cultures were incubated at 12 °C for 72 h under static conditions. Three independent experiments were performed for both mixed and monoculture conditions. At 0, 3, 6, 8, 24, 48, and 72 h, samples were collected to measure pH (pH meter Altronix TPX I, Altronix, Brooklyn, NY, USA) and microbial viability. Serial dilutions were prepared in buffered peptone water and plated on MRS agar (30 °C, 48 h) and MacConkey agar (Britania, Buenos Aires, Argentina; 30 °C, 24 h) for LAB and *E. coli* NCTC 12900 enumeration, respectively.

### 2.4. LAB–EHEC Interaction in Biofilm Under Technological Conditions at 12 °C

The exclusion, competition, and displacement assays, used to investigate the behavior of each LAB strain able to prevent or disrupt EHEC biofilms, were carried out as described by Cisneros et al. [20]: at 12 °C, including a pre-incubation time at 25 °C. In order to eliminate lipids and all residues that could be present on the surfaces, prior to each assay, new stainless steel (SS) chips of 1 × 1 cm or 5 × 7 cm (for proteomic assay) were immersed in acetone during 30 min and thereafter rinsed carefully with distilled water and immersed in 1M NaOH for 1 h. Thereafter, they were rinsed with distilled water and left to dry at ambient temperature. Finally, SS chips were sterilized by autoclaving (121 °C, 20 min).

#### 2.4.1. Exclusion Assay

The experiment aimed to determine whether LAB biofilms could hinder *E. coli* adhesion and biofilm formation; for this purpose, MES was inoculated with LAB cultures adjusted to 10^7^–10^8^ CFU/mL. One milliliter of the LAB suspension was dispensed into each well of a 24-well polystyrene microplate containing 1 × 1 cm stainless steel (SS) chips. The plates were first incubated at room temperature for 6 h to promote bacterial adhesion, followed by incubation at 12 °C for an additional 36 h, resulting in a total incubation period of 42 h for biofilm development. After this step, the supernatants were removed, and each well containing SS chips with pre-established LAB biofilms was overlaid with 1 mL of *E. coli* culture (10^7^–10^8^ CFU/mL) prepared in MES. The plates were then incubated at 12 °C for 24 h. Subsequently, the chips were transferred into PBS and sonicated to detach sessile cells. The viability of both LAB and *E. coli* O157:H7 populations was quantified as log CFU/cm^2^ of chip by plating on MRS and MacConkey agar, respectively. Control assays consisted of *E. coli* or LAB biofilms formed in the absence of LAB or EHEC, respectively.

#### 2.4.2. Competition Assay

One milliliter of each LAB and EHEC culture grown in MES (10^7^–10^8^ CFU/mL) was inoculated into individual wells of a 24-well polystyrene microplate, each containing a 1 × 1 cm stainless steel (SS) chip. The plates were incubated at 12 °C for 48 h, with the culture medium renewed after 24 h. Following incubation, the SS chips were carefully removed, rinsed to eliminate non-adherent cells, and transferred to Falcon tubes containing PBS. Biofilm cells were detached by sonication, and sessile populations were enumerated by plating on selective agar media, as described in Section 2.4.1. In addition, 48 h biofilms formed by EHEC and LAB individually were included as respective control samples.

#### 2.4.3. Displacement Assay

NCTC 12900 biofilm formation was carried out for 24 h. Thereafter, 1 mL of Lactic Acid Bacteria culture (10^7^–10^8^ log CFU/mL of MES) was inoculated onto the previously formed *E. coli* NCTC 12900 biofilm and incubated at 12 °C for 24 h. A 24 h and 48 h old biofilm of LAB and EHEC, respectively, were used as controls.

### 2.5. Inhibitory Action of LAB Culture Supernatants on E. coli NCTC 12900 Biofilm

#### 2.5.1. Culture Supernatant Recovery

Single LAB and mixed LAB+EHEC planktonic cultures in MES were carried out as described in 2.3 and incubated for 24 and 48 h at 12 °C. Thereafter the planktonic cultures were centrifuged at 8500× *g* for 14 min. The supernatants were recovered, sterilized by filtration using 0.22 µm-pore-size filters (Steritop GP, Biopore, Buenos Aires, Argentina), and stored at −20 °C until used.

#### 2.5.2. EHEC Biofilm Formation in the Presence of Culture Supernatants

The supernatants (LAB or LAB+EHEC) obtained at 24 and 48 h were used. SS (1 × 1 cm) coupons were placed in a 24 well microplate, 1 per well. One mL of MES inoculated with 8 log CFU/mL of *E. coli* NCTC 12900 and 1 mL of obtained supernatants were seeded onto each SS coupon in the microplate. A control with 1 mL of PBS instead of culture supernatant was carried out. After 6 h at 25 °C, the plate was incubated at 12 °C, to complete the 24 h. Afterward, the chips were recovered and processed accordingly for cell quantification as described in 2.4.2.

#### 2.5.3. EHEC Preformed Biofilms Exposed to LAB Culture Supernatants

Eighty μL of each of the obtained supernatants (from LAB or LAB+EHEC supernatants) (or PBS as control) were applied onto 24 h old EHEC biofilm formed on SS coupons, as described in Section 2.4, and incubated for 6 h at 12 °C. The viability of the sessile EHEC population was quantified by plating in McConkey agar, as described in Section 2.4.

### 2.6. Live/Dead Assay for Sessile E. coli NCTC 12900 Differentiation

The kit FilmTracer LIVE/DEAD Biofilm Viability^®^ (Invitrogen Thermo Fisher Scientific, Waltham, MA, USA) was used for direct viable and total counts of sessile NCTC 12900 cells. The two BacLight stains, SYTO 9^®^ and propidium iodide (PI), were mixed together (2 μL + 2 μL) and diluted with 50 μL of ultrapure water. The stock solution was stored at −20 °C in the dark. The fluorochrome SYTO 9^®^ penetrates all cells, whereas PI only penetrates damaged cells. A volume of 50 μL of the BacLight stock solution was added to the biofilm sample. Samples were incubated for 15 min at 4 °C in dark conditions. Afterward, the stained sample was mounted in BacLight mounting oil. This assay allowed us to qualitatively visualize via confocal laser scanning microscopy damaged/dead and live cells. Samples were visualized using a Zeiss LSM 800 confocal laser scanning microscope (CLSM)(Zeiss, Oberkochen, Germany) using 488 nm and 561 nm lasers at 0.2% intensity for each channel (IMMCA-CONICET-UNT-SIPROSA). Z-stacks of biofilms (12 slices, (2.53 μm) were acquired using 63 × objective (703 × 703 pixels), an LSM scan speed of 4, and a pinhole set at 1.00 AU/45 μm. Fluorescence emissions from each channel were captured individually on different tracks, using a Multialkali-PMT detector with a gain of 550 V and 600 V for the 488 nm and 561 nm channels, respectively. Maximum Intensity Projections (MIPS) were created using the Ortho tool in the Zeiss system (ZEN2.6 blue edition). Viable and non-viable cells were fluorescent green and red, respectively. Figures from confocal images were prepared using ImageJ (1.48v version).

### 2.7. Proteomic Studies

To unravel part of the mechanisms carried out by *P. pentosaceus* CRL 2145 during EHEC biofilm inhibition, differential protein expression was analyzed by means of shotgun bottom-up proteomics, a technology that can provide more information about the metabolic response of LAB to the presence of this pathogen.

The assay was carried out at 12 °C using the competition as a strategy of interaction. Differential expression of *P. pentosaceus* CRL 2145 proteins after 18 h of interaction with EHEC under biofilm growth was assessed. Under this condition, the viability of the pathogen begins to decline due to the presence of LAB. A single biofilm was obtained per each microorganism under identical conditions. Thereafter, the resulting cultures were collected and mixed so as to obtain a mixed sample whose proteome denoted both microorganisms separately grown under sessile condition (control). The goal was to identify LAB proteins presenting differential expression in mixed biofilms with EHEC (Condition 1) versus monoculture biofilms (Condition 2: control). To avoid bias from unequal protein input, sessile LAB and *E. coli* cells grown separately were mixed, prior to lysis, in the same ratio (LAB: EHEC) observed in Condition 1. This ensured comparable extraction conditions across assays, allowing differences to reflect biological interactions rather than technical artifacts (Figure S1).

#### 2.7.1. Cell Collection

LAB and EHEC were grown individually (controls) and as mixed cultures under biofilm conditions. For sessile proteome collection, larger coupons (5 × 7 cm) were required in order to obtain sufficient biomass for LC-MS analysis. The biofilm assay was conducted following the same protocol and bacterial concentrations as described for the competition assay (Section 2.4.2), with only the working volumes adjusted to ensure equivalent initial bacterial loading and comparable sessile populations at the starting point. For the individual biofilms, 20 mL bacterial suspension of each bacterium (ON cultures of *E. coli* and *P. pentosaceus*) in MES were prepared, reaching a concentration of 8 log CFU/mL. Subsequently, 5 mL of this suspension (separately) and 5 mL of MES were inoculated onto each Petri dish previously prepared with one SS coupon (5 × 7 cm) (four plates/each condition). Parafilm^®^ was used to seal each plate that was then stored 2 h at room temperature and finally incubated at 12 °C for 18 h. Thereafter, each chip was immersed in 30 mL of 0.1 M Tris-HCl, pH 7.5, and then transferred to a new plate with 10 mL of 0.1 M Tris-HCl, pH 7.5, and vigorously scraped with a sterile spatula to detach the maximum number of cells, which were collected in a conical base tube containing an adequate volume and homogenized in a Vortex for 5 min. Sessile cell counting was carried out on 100 μL of this mixture. A centrifugation at 8000× *g* and 10 °C for 15 min allowed for the collection of cells that were washed twice with 1 mL of 20 mM Tris-HCl, pH 7.5, containing 5 mM EDTA and 5 mM MgCl_2_. The resulting supernatants were discarded, while pellets were kept at −20 °C until proteome recovery. Per each condition, three biological replicates were undertaken. Cells from mixed biofilms were obtained by preparing a MES suspension (8 log CFU/mL) of LAB and EHEC strains from ON cultures. Five mL of each suspension was simultaneously inoculated onto plates containing the SS chip. Parafilm^®^ was used to seal the plates that were stored as described for the control. The resulting cells were collected as previously described. To avoid differences in the protein enrichment of each microorganism that could affect proteomic comparisons, the efficiency of cell disruption between mono- and co-cultures was standardized by mixing before lysis the cell pellets obtained from *P. pentosaceus* and *E. coli* coming from their individual biofilm. Such mixture represented the control (both bacteria cells grown separately in biofilm) (Figure S1). The proteomic profile of *P. pentosaceus* CRL 2145 grown as single-species biofilm (control) was compared with that mixed with *Escherichia coli* NCTC 12900 biofilm. Cell-free extracts (CFE) were obtained by cell lysis, applying sonication and glass beads disruption [20].

#### 2.7.2. Protein Concentration and Digestion

The proteomics facility required that each biological replicate of CFE contain exactly the same total protein content, which allowed for accurate normalization and robust assessment of differential protein expression. CFE samples were mixed with two volumes of acetone. The solution was strongly mixed and kept for 16 h at −20 °C. Thereafter, it was centrifuged for 16 min at 14,600× *g*. The resulting supernatant was discarded. The pellet was dried for 1–3 min at 95 °C. Thereafter, the samples were concentrated by SDS-PAGE [20]. The staining of the gels was carried out using Coomassie Blue Safe (Bio-Rad, Hercules, USA), following the manufacturer’s instructions. After the staining process with deionized water, bands were cut and stored at 4 °C. Protein digestion and identification analyses through mass spectrometry (MS) were carried out at the CEQUIBIEM Proteomics Centre (QB-FCEN-UBA/IQUIBICEN-CONICET). The digests were analyzed using nanoLC-MS/MS in a Thermo Scientific QExactive Mass Spectrometer coupled to a nanoHPLC EASY-nLC 1000 (Thermo Scientific, Dreieich, Germany) [25]. Briefly, LC-MS/MS analysis was performed using a reverse phase column (C18, 2 µm, 100A, 50 µm × 150 mm) Easy-Spray Column PepMap RSLC (P/N ES902) coupled to Thermo Scientific Q-Exactive with Electro Spray Ionization (EASY-SPRAY, Thermo Scientific, Dreieich, Germany), a high collision dissociation cell (HCD) for fragmentation, and an Orbitrap analyzer (Q-Exactive, Thermo Scientific, Dreieich, Germany).

#### 2.7.3. Analysis of MS Data

Proteome Discoverer software (version 2.2 Thermo Scientific) was used for processing QExactive raw data which were searched against the *Pediococcus pentosaceus* (strain ATCC 25,745 CCUG 21,536 LMG 10,740 183, https://www.uniprot.org/proteomes/UP000000773 (accessed on 23 March 2022) protein sequences database with trypsin specificity and a maximum of one missed cleavage per peptide. Proteome Discoverer searches were performed with a precursor mass tolerance of 10 ppm and product ion tolerance of 0.05 Da. Static modifications were set to carbamidomethylation of Cys, and dynamic modifications were set to oxidation of Met and N-terminal acetylation. Protein hits were filtered for high confidence peptide matches with a maximum protein and peptide false discovery rate of 1%, calculated by employing a reverse database strategy.

Per each of the three biological replicates, the areas were calculated and later normalized. Data were processed using the Perseus program (Max Planck Institute of Biochemistry, 1.5.5.3 version), allowing for a robust statistical analysis. According to the compared samples, different scatter plots were performed. Log *p*-value (–Log Student T-test *p*-value A_B) on the y-axis vs. Student T-test Difference A_B on the *x*-axis were plotted for each pair of samples. Differentially expressed proteins were those appearing in the volcano plot with a fold change greater than 2 (less than −1 or greater than 1 on the *x*-axis of the graph) and a *p*-value below 0.05 (above 1.3 on the *y*-axis of the graph) [20]. Then, proteins with a fold change expression (FC) > 2 and a *p*-value < 0.05 were considered significant.

#### 2.7.4. Bioinformatic Analysis

The databases Universal Protein Resource (UniProt) and the Eggnog online framework were used to perform the functional classification of the identified proteins, to detect the clusters of orthologous groups (COGs) [26]. The STRING database, using the accession numbers of differentially expressed proteins, was used to investigate protein–protein interactions [25]. In the STRING analysis, proteins were considered functionally associated based on documented or predicted interactions [27]. The network was constructed by linking each protein to other differentially expressed proteins herein identified under the studied conditions. In this network, each protein is represented as a node, and the connecting lines indicate functional associations. The thickness of these lines reflects the confidence of the interaction according to all prediction sources integrated by STRING (curated databases, experimental data, gene neighborhood, fusion, co-occurrence, text mining, co-expression, and homology). Networks were constructed exclusively from the identified differentially expressed proteins (*p* < 0.05; FC > 2), without adding external protein partners or applying manual adjustments. A confidence level of 0.4 was used.

#### 2.7.5. Data Availability

The mass spectrometry proteomics data have been deposited to the ProteomeXchange Consortium via the PRIDE partner repository [28,29,30] with the dataset identifier PXD067300.

### 2.8. Statistical Analyses

In all cases, at least two independent biological experiments were performed, and in several instances (e.g., biofilm assays and proteomic analyses), three or four independent biological replicates were included. Within each biological replicate, data points were measured in technical triplicates. As indicated in the figures, results are presented as mean ± standard error (SE). The R-medic statistical software (1.0 version) available online [31] was used to conduct the statistical analyses. Depending on the nature of the data, one-way analysis of variance (ANOVA), with the Tukey test, or the Kruskal–Wallis test (non-parametric) were applied to assess the statistical differences, which were considered significant for *p*-values < 0.05. Significance (*p* < 0.05) was indicated either by error bars or by different letters denoting significant differences.

## 3. Results

### 3.1. LAB–EHEC Interaction in the Meat Experimental System Under Planktonic Conditions

**Figure 1:**
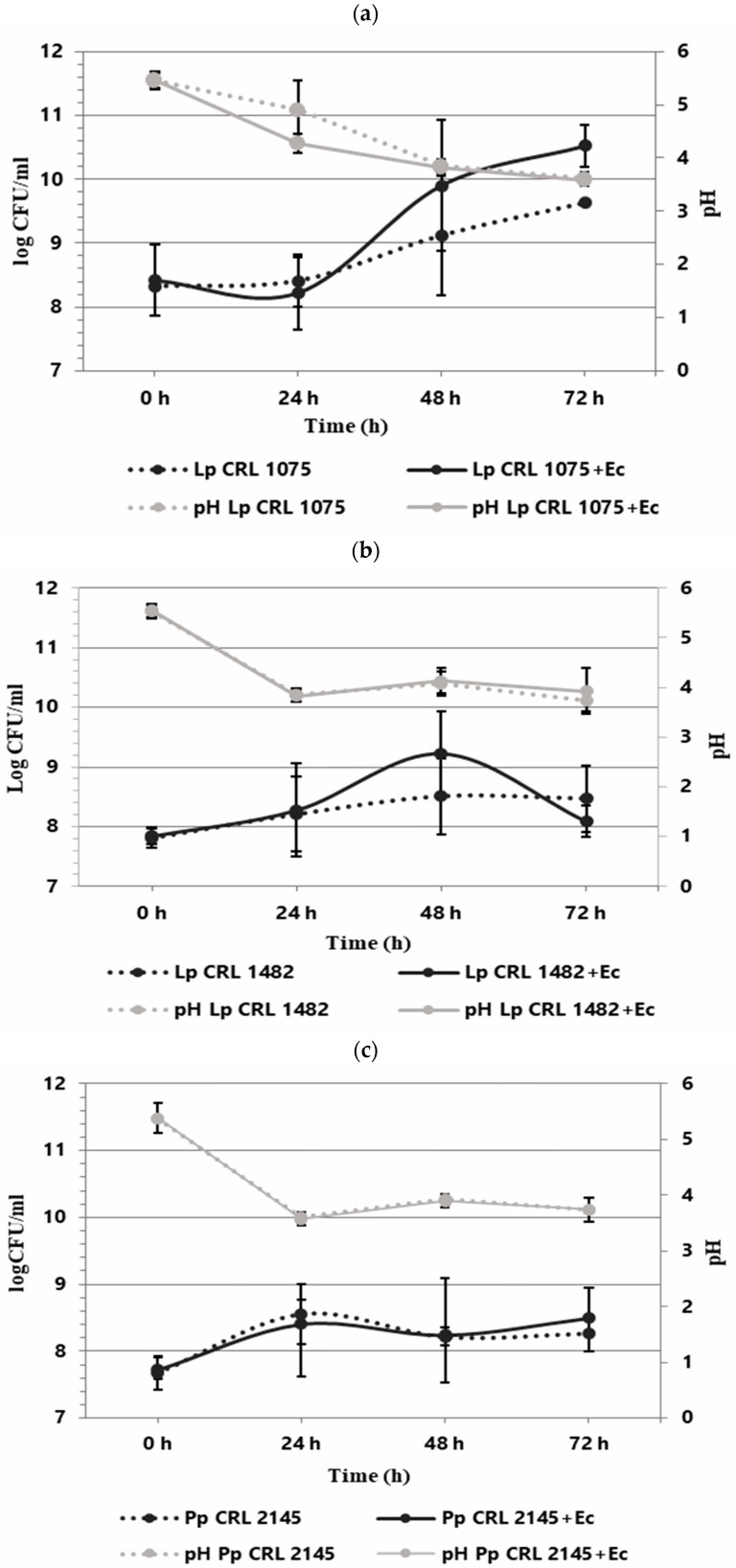
Growth kinetics (logCFU/mL) of LAB individually and in co-culture with *E. coli* NCTC 12900 (LAB+Ec) in the Meat Experimental System (MES) at 12 °C. pH (gray lines). Individual growth (dotted lines) and co-culture growth (solid lines). Individual and co-culture growth of (**a**) *L. plantarum* CRL 1075, (**b**) *L. plantarum* CRL 1482, and (**c**) *P. pentosaceus* CRL 2145.

**Figure 2:**
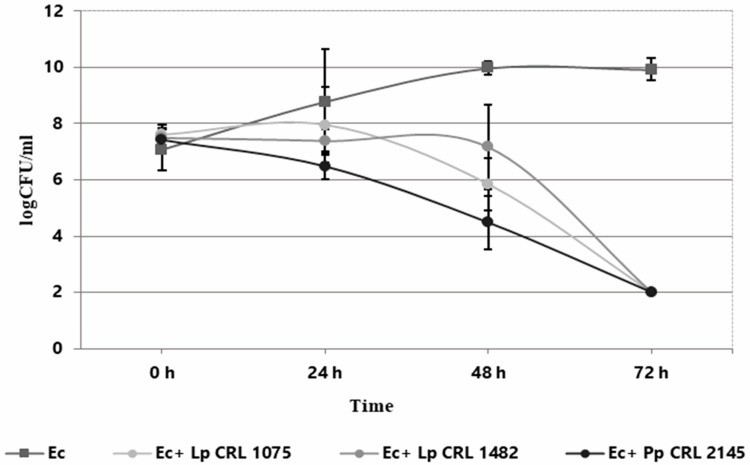
Growth kinetics (log CFU/mL) of *E. coli* NCTC 12900 as individual (Ec) and co-culture (Ec+LAB) with *L. plantarum* CRL 1075 (Lp CRL 1075), *L. plantarum* CRL 1482 (Lp. CRL 1482), and *P. pentosaceus* CRL 2145 (Pp. CRL 2145) in the Meat Experimental System (MES) at 12 °C during 72 h.

The individual and co-culture growth of LAB strains and *E. coli* O157:H7 NCTC 12900 was studied in the Meat Experimental System (MES) to evaluate planktonic interactions. As shown in Figure 1, the three LAB strains exhibited different growth behavior when cultured individually. *L. plantarum* CRL 1075 had a prolonged lag phase, entering into exponential growth at 24 h and reaching its maximum cell concentration at 72 h (9.63 log CFU/mL). *L. plantarum* CRL 1482 and *P. pentosaceus* CRL 2145 showed linear growth, with the former maintaining stable counts (8.28–8.47 log CFU/mL) and CRL 2145 showing a slight decline at the end of incubation. All LAB strains caused a notable pH drop, with *L. plantarum* CRL 1075 producing the strongest acidification. When cultured alone, EHEC reached its exponential peak at 48 h (9.97 log CFU/mL), entering the stationary phase thereafter (Figure 2). In co-culture, LAB strains followed similar growth kinetics to their pure cultures. *L. plantarum* CRL 1075 showed improved viability at 48 and 72 h, increases of 0.78 and 0.89 log units, respectively. *P. pentosaceus* CRL 2145 grew similarly to its control, and *L. plantarum* CRL 1482 reached its maximal growth at 48 h (0.7 log units increase), then declined to initial levels by 72 h. Acidification was not affected by EHEC co-cultivation, with final pH values between 3.5 and 4.0. EHEC growth was significantly inhibited in co-culture with all three LAB strains, particularly after 20 h. At 72 h, EHEC counts dropped to <100 CFU/mL in all co-cultures. *P. pentosaceus* CRL 2145 showed the fastest inhibition, while *L. plantarum* CRL 1482 was slower, showing significant reduction only after 48 h. Overall, EHEC viability was reduced by >5 log CFU/mL compared to the initial inoculum and by >8 log CFU/mL relative to its pure culture (Figure 2). These results highlight the strong antagonistic effect of all three LAB strains against EHEC under planktonic and low-temperature conditions. The next step was to evaluate whether this effect persisted under sessile conditions.

### 3.2. LAB Performance During the Interaction with EHEC in Biofilm Under Meat Technological Conditions

In this study, the biofilm populations of the LAB strains were not significantly affected by the presence of *E. coli* NCTC 12900, with the exception of *L. plantarum* CRL 1482, which exhibited a reduction in biomass under the displacement strategy (Table 1). Overall, the sessile LAB populations remained stable across the three interaction strategies evaluated.

### 3.3. Inhibitory Activity of LAB Culture Supernatants on E. coli NCTC 12900 Biofilm on SS Chips at 12 °C

**Figure 3:**
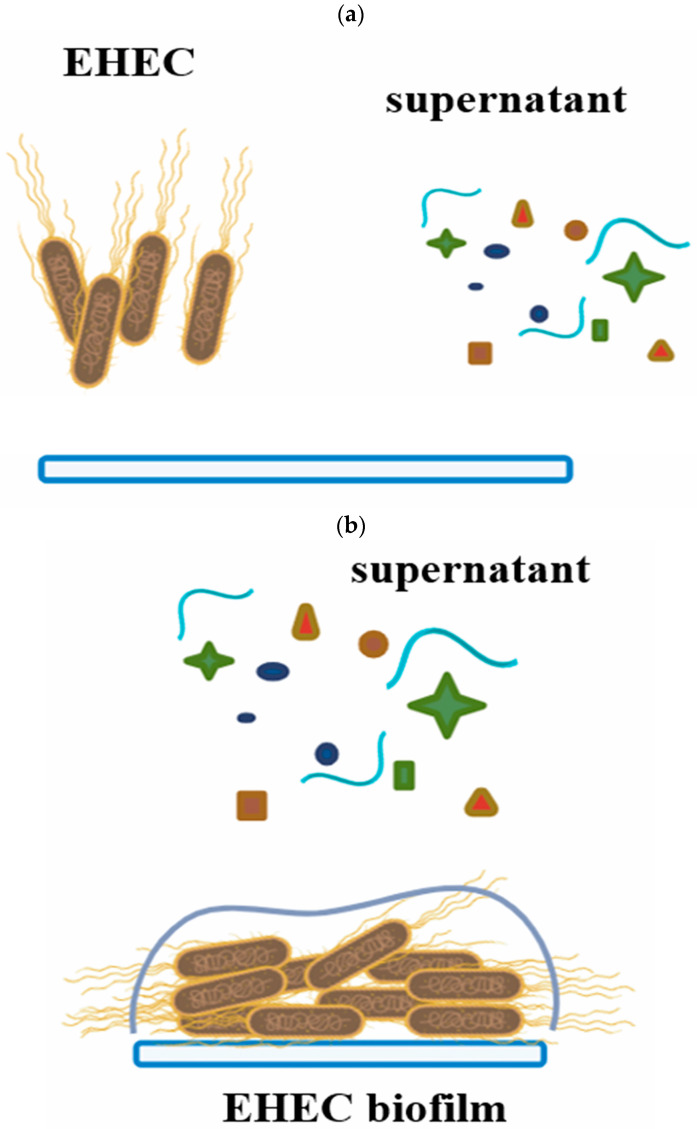
Schematic representation showing how the effects of supernatants from pure LAB cultures or mixed LAB+EHEC cultures on EHEC biofilms were analyzed. (**a**) Simultaneous inoculation of EHEC with supernatants on stainless steel chips. (**b**) Addition of supernatants to a 24 h pre-formed EHEC biofilm.

The antimicrobial activity of 24 and 48 h supernatants from pure LAB cultures and mixed cultures (LAB+EHEC) was evaluated on *E. coli* NCTC 12900 biofilm formed on SS chips at 12 °C. Two assays were conducted: (i) the supernatants, from pure (LAB) or mixed cultures (LAB+EHEC) were co-inoculated with an *E. coli* suspension (1 × 10^8^ CFU/mL) on SS chips and analyzed after 24 h incubation, and (ii) the supernatants (LAB or LAB+EHEC) were applied onto an already established 24 h old *E. coli* biofilm on SS chips at 12 °C and analyzed after 6 h (Figure 3).

#### 3.3.1. Effect of Simultaneous Inoculation of Culture Supernatants (LAB; LAB+EHEC) on EHEC Biofilm Formation

**Figure 4:**
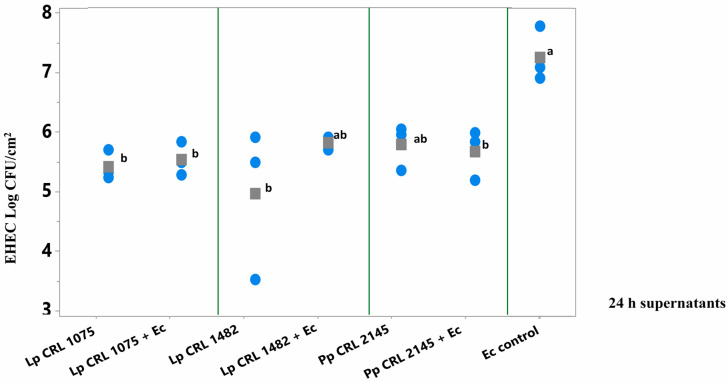
*E. coli* NCTC 12900 sessile cells (log CFU/cm^2^) after the simultaneous supernatant addition on SS chips and analyzed after 24 h of incubation at 12 °C. Supernatants from 24 h LAB cultures or mixed (LAB+EHEC) co-cultures; Control (Ec control): EHEC sessile cells (log CFU/cm^2^) with addition of Meat Experimental System (MES). Assayed LAB strains: *L. plantarum* (Lp) Lp CRL 1075, Lp CRL 1482 and *P. pentosaceus* (Pp) CRL 2145 and mixed cultures (LAB+Ec). Different letters indicate statistically significant differences (*p* < 0.05).

As shown in Figure 4, pure culture supernatants of *L. plantarum* CRL 1075 and CRL 1482 (at 24 h) significantly reduced (*p* < 0.05) pathogen biofilm formation. Specifically, reductions of 1.86 log CFU/cm^2^ (24 h) were observed for CRL 1075, while CRL 1482 achieved reductions of 2.75 log CFU/cm^2^ (24 h). The mixed culture supernatants (LAB+EHEC) from *L. plantarum* CRL 1075 and *P. pentosaceus* CRL 2145 also significantly reduced EHEC sessile biomass, by approximately 1.5 to 1.7 log units. Notably, the 24 h mixed supernatant from *L. plantarum* CRL 1482 exhibited the strongest inhibitory effect, achieving a reduction of 2.4 log CFU/cm^2^ (Figure 4). No significant differences in the reduction in EHEC sessile biomass was observed between 24 and 48 h culture supernatants nor between pure and mixed supernatants (Figure S2).

#### 3.3.2. Effect of Culture Supernatants (LAB and LAB+EHEC) on Pre-Formed EHEC Biofilm on SS Chips

**Figure 5:**
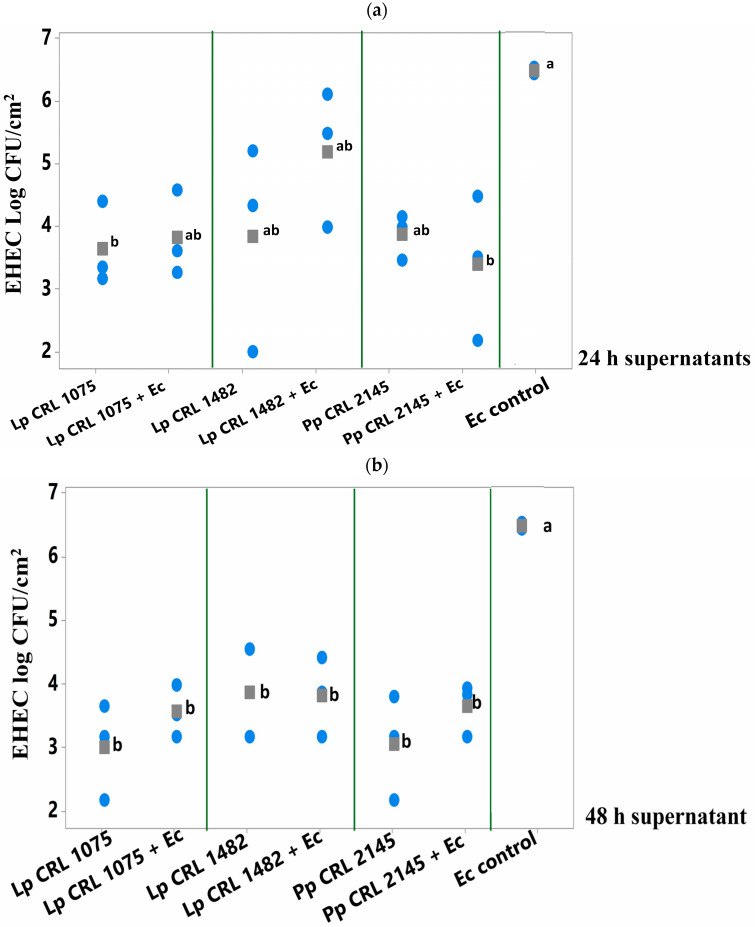
*E. coli* NCTC 12900 sessile cells (log CFU/cm^2^) after being treated or not (control) with (**a**) 24 h supernatants or (**b**) 48 h supernatants from pure (LAB) and mixed cultures (LAB+EHEC) and analyzed after 6 h of incubation at 12 °C. Control of *E. coli* NCTC 12900 (Ec); *L. plantarum* (Lp.) Lp. CRL 1075, Lp. CRL 1482, *P*. *pentosaceus* (Pp) CRL 2145. Different letters indicate statistically significant differences (*p* < 0.05).

Among the 24 h supernatants, only that from *L. plantarum* CRL 1075 and the mixed culture supernatant from *E. coli* + *P. pentosaceus* CRL 2145 significantly break up (*p* < 0.050) the preformed *E. coli* biofilm after 6 h of treatment (Figure 5a). In contrast, the 48 h supernatants of the three LAB strains—both from pure and mixed cultures—produced a significant reduction in the pathogen’s biofilm, achieving decreases of 2.6 to 3.5 log units compared to the *E. coli* biofilm control (Figure 5b). The greatest reductions were observed with the 24 h supernatant from the mixed *P. pentosaceus* CRL 2145 + *E. coli* culture (–3.0 log CFU/cm^2^) and the 48 h supernatant from *L. plantarum* CRL 1075 (–3.5 log CFU/cm^2^). This strategy showed higher inhibitory effect on *E. coli* biofilm than the simultaneous supernatants addition (Section 3.3.1).

*P. pentosaceus* CRL 2145 emerged as the most robust antagonistic strain, as its population remained stable during co-cultivation with EHEC under both planktonic and biofilm conditions. For this reason, this strain was selected for subsequent studies.

### 3.4. Live/Dead Staining

**Figure 6:**
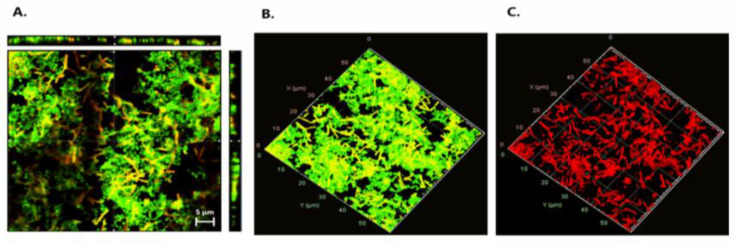
Live/dead staining. (**A**) Orthogonal representation of the mixed biofilm with live and damaged/dead cells. (**B**) Three-dimensional representation of live cells. (**C**) Three-dimensional representation of damaged/dead cells. Live bacteria with intact membranes fluoresce green (SYTO 9), while dead or membrane-compromised cells fluoresce red (propidium iodide), as detected using the FilmTracer™ LIVE/DEAD^®^ Biofilm Viability Kit.

To assess whether *E. coli* cells were damaged by the effect of LAB during mixed-species biofilms, a live/dead staining assay was performed using *P. pentosaceus* CRL 2145 as the selected strain (Figure 6). Figure 6A presents an orthogonal (sectional) view of the biofilm, showing three planes—xy, xz, and zy—which provide an estimation of biofilm thickness and population density, as well as the spatial distribution of live and damaged/dead cells. Figure 6B,C show the three-dimensional structure of the biofilms. Observation through the red fluorescence filter (Figure 6C) revealed that the majority of the non-viable (red-stained) cells corresponded to *E. coli* bacilli, indicating a predominance of dead *E. coli* cells within the biofilm. While the *Pediococcus* population also contained damaged/dead cells, these were present at a noticeably lower proportion. As shown in Figure 6A, most of the damaged/dead cells were located within the microcolonies of the mixed biofilms.

### 3.5. Differential Proteomic Analysis of Sessile P. pentosaceus Cells in Mixed Biofilm with EHEC NCTC 12900

In our study, 346 *P. pentosaceus* proteins were identified under biofilm conditions after 18 h of co-incubation with EHEC, representing approximately 20% of the total reference proteome, which consists of 1755 proteins (https://www.uniprot.org/proteomes/UP000000773) (accessed on 20 September 2025).

The proteomic profiles of *P. pentosaceus* in a mixed biofilm with or without EHEC on SS chips was analyzed. A total of 162 differentially expressed proteins were successfully identified (96.3% overexpressed and 3.7% under-regulated) (Table S1). Resulting from the statistical analysis (FC > 2 and *p* value < 0.05), these proteins were observed to belong to various cellular functions and were classified into functional categories based on the COG database.

#### 3.5.1. *P. pentosaceus* Proteins Synthetized in Higher Amounts During Mixed Biofilm Growth

**Figure 7:**
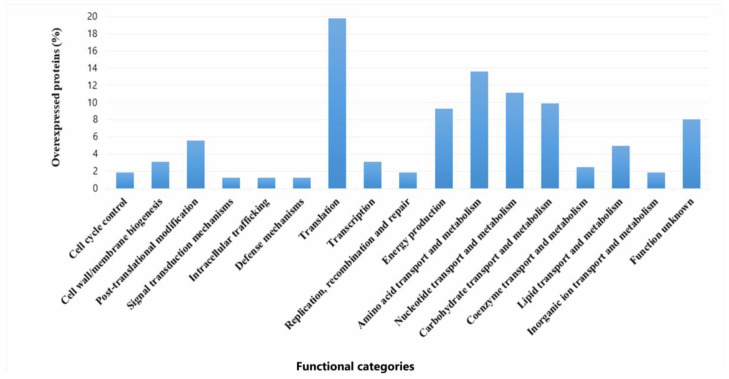
Functional categories and percentages of positively regulated proteins in sessile *P. pentosaceus* CRL 2145 cells during their growth with EHEC on SS chips at 12 °C.

Overexpression levels spanned from 2- to 112.9-fold relative to the individual sessile state. (Table S1). A major part of the upregulated proteins belonged to translation (19.6%), metabolism of amino acids (13,5%), nucleotides (9,8%), carbohydrates (13,5%), and energy production (9,2%). Proteins with unknown function made up 8% (Figure 7). Among the upregulated proteins, several stand out due to their high expression levels (Fold Change (FC) ranging from 70 to 113). Notably, a protein containing a cystathionine beta-synthase (CBS) domain (Q03DZ5, FC: 112.9), which is found in both cytosolic and membrane proteins involved in various functions (such as metabolic enzymes, kinases, and channels). Also highlighted are valine–tRNA ligase (Q03EP0, FC: 110.9), which is involved in the attachment of the amino acid valine to its corresponding tRNA, and the preprotein translocase YajC (Q03ER3, FC: 70.9), which plays a role in bacterial secretion systems. Among the proteins with expression levels ranging from 10.0 to 30.0, several are involved in amino acid metabolism. Notable examples include two peptidase enzymes: proline iminopeptidase (Q03H47, FC: 29.9), a dipeptidase (Q03E86, FC: 16.7), and an enzyme involved in amino acid biosynthesis, N-succinyl-L-diaminopimelate desuccinylase (Q03HV4, FC: 13.9). Proteins associated with carbohydrate transport and metabolism were also overexpressed, such as glucose-6-phosphate isomerase (Q03EI3, FC: 17.4), glucose-6-phosphate 1-dehydrogenase (Q03ER4, FC: 16.9), triosephosphate isomerase (Q03GW6, FC: 11.7), and sugar acid phosphatase (Q03EV4, FC: 11.8). In lipid metabolism, a fatty acid transport protein (Q03FV7, EC: 10.3) stood out, while in nucleotide transport and metabolism, CTP synthase (Q03DY0, EC: 17.4) was notably upregulated. In the category of energy production and conversion, key enzymes included the malolactic enzyme (Q03DT4, EC: 13.5) and D-lactate dehydrogenase LdhA (Q03FT7, EC: 11.6). Within the defense mechanisms category, a notable protein was the ATPase component of an antimicrobial peptide transport system (Q03DF8, EC: 22.8). In the translation, ribosomal structure, and biogenesis category, elongation factor Tsf (Q03FT5, EC: 14.4) was highlighted. In the transcription category, the transcription/antitermination factor NusA (Q03FS6, EC: 25.3) was particularly relevant. Additionally, an endopeptidase PepO (Q03HC3, EC: 19.0) and a chaperonin HslO (Q03E07, EC: 16.3) were also identified as overexpressed.

#### 3.5.2. *P. pentosaceus* Under-Expressed Proteins in Mixed Biofilm

**Figure 8:**
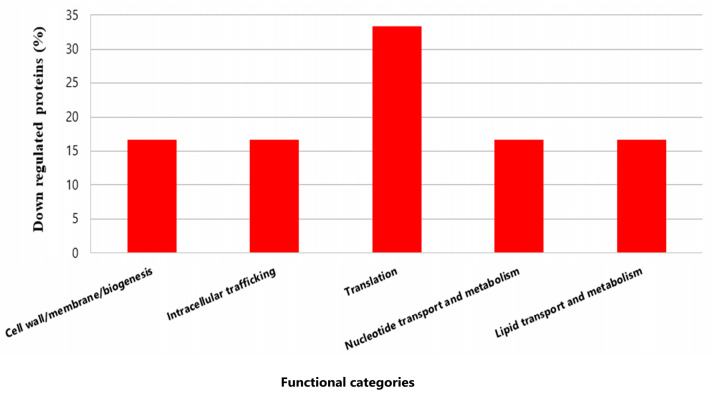
Functional categories and percentages of negatively regulated proteins in sessile *P. pentosaceus* CRL 2145 cells during their growth with EHEC on SS chips at 12 °C.

Only six proteins from *P. pentosaceus* CRL 2145 were expressed in lower amounts during the interaction under biofilm conditions with EHEC. Notably, the Sec translocase complex subunit A1 exhibited a much lower expression level (Q03GZ8; FC: −528.0) (Figure 8).

In summary, during the growth of *P. pentosaceus* and EHEC in biofilm, the LAB predominantly overexpressed proteins. Very few proteins were downregulated, suggesting a metabolic activation of the bacterium in the presence of the pathogen.

#### 3.5.3. Interaction Among Differentially Expressed Proteins in Sessile *P. pentosaceus* CRL 2145 Cells in Mixed Biofilms

**Figure 9:**
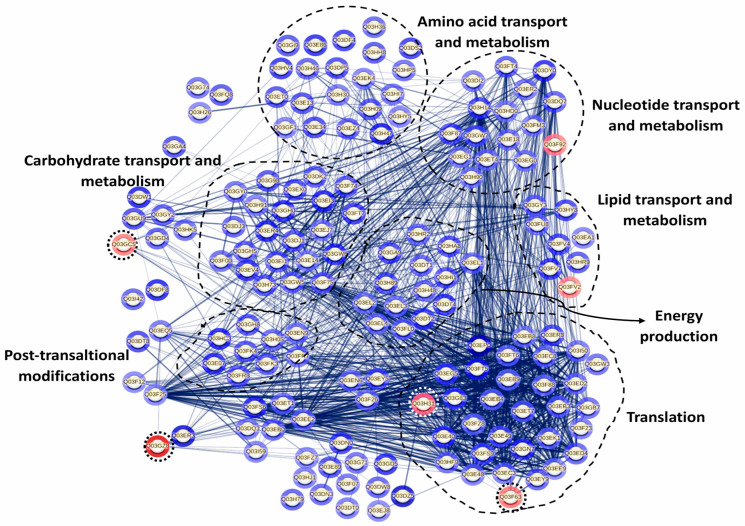
Protein interaction network in *P. pentosaceus* CRL 2145 co-cultured with *E. coli* NCTC 12900 under sessile conditions. Nodes and edges represent proteins and their interactions, respectively, The thickness of the edges provides the strength of the different interactions. Only proteins presenting a statistically different expression were considered for this analysis. The relative expression level is indicated by the color intensity in gray scale of halos around each node: the few under-regulated proteins are surrounded by a dotted circle; the rest are overexpressed proteins. STRING v11.5 was used for this analysis (see https://string-db.org/cgi/network?taskId=bLD26PBiWOxP&sessionId=bkCBxBXFa4YE, accessed 21 October 2025).

Since proteins do not function independently but rather coordinate with one another to carry out a series of reactions essential for their biological roles, a protein–protein interaction (PPI) network was constructed using the STRING database. This analysis aimed to identify potential interaction networks among the differentially expressed proteins of *P. pentosaceus* CRL 2145 within the mixed biofilm with *E. coli* NCTC 12900 (Figure 9). PPI results revealed that a total of 15 proteins did not interact with each other, while 147 proteins were interconnected. Additionally, four proteins exhibited pairwise interactions. When the LAB strain grew in biofilm with EHEC, most of the LAB proteins with strong interactions were associated with key cellular processes, including cell biosynthesis, protein translation, proteolysis, nucleotide biosynthesis, carbohydrate metabolism, and oxidation–reduction processes. On the other hand, only six proteins were downregulated. Among them, the most strongly repressed protein was the SecA1 subunit of the protein translocase complex, which is involved in intracellular trafficking. Notably, this protein showed strong interactions with other proteins associated with translation and cell biosynthesis (Figure 9).

## 4. Discussion

### 4.1. Physiologic Studies

Given the potential use of LAB strains as biocontrol agents, this study evaluated the capacity of selected LAB isolates to inhibit *Escherichia coli* NCTC 12900 in a meat system (MES) by monitoring the growth kinetics of LAB–EHEC co-cultures at 12 °C. This experimental approach has previously been used by other authors to evaluate interactions between beneficial and pathogenic microorganisms; however, most studies have relied on commercial media without considering the influence of the culture medium, whereas in this study, a meat-based medium extensively validated by our group was employed [32,33]. As shown previously under biofilm conditions [20], in planktonic cultures, the three LAB strains negatively affected EHEC growth in the meat environment after 48 h of incubation, showing the highest inhibitory potential at 72 h, with pathogen cell counts falling below 100 CFU/mL. These results demonstrate the capacity of LAB strains to sense in liquid medium the presence of a competitor and mount protective measures to guarantee its survival and growth.

Although previous screening assays indicated that neither LAB culture supernatants nor the lactic acid produced by LAB inhibited the growth of *E. coli* NCTC 12900 in agar plate tests [17,19], it was nevertheless of interest to evaluate their effect on biofilm formation on an inert surface. In this context, the use of non-treated LAB supernatants was intentionally selected to explore their potential as a biological strategy for surface decontamination, considering that such supernatants could contain a complex mixture of active metabolites acting synergistically. Remarkably, the non-treated supernatants derived from both LAB monocultures and LAB–EHEC co-cultures effectively inhibited EHEC biofilm formation. Furthermore, their application to pre-established EHEC biofilms resulted in a significant reduction in sessile populations, indicating a potential biofilm-disrupting activity. These findings suggest that LAB supernatants harbor bioactive molecules—most likely organic acids, antimicrobial peptides, or hydrolytic enzymes—that mediate antagonistic interactions on stainless steel surfaces. Future studies using neutralized and enzymatically treated supernatants, coupled with compositional and secretomic analyses, will be essential in order to elucidate the specific components and mechanisms responsible for this inhibitory effect. Overall, these results support the concept that whole, non-treated LAB supernatants may represent a promising and sustainable alternative for the biological control of pathogenic biofilms in food processing environments.

Combining our results with those earlier reported [20], it can be stated that the most effective strategy of EHEC inhibition was competition, yielding reductions between 2.8 and 6 log CFU/cm^2^, followed by exclusion, with reductions between 2.8 and 3.5 log CFU/cm^2^ and the treatment of pre-established biofilms with LAB culture supernatants, which reduced by 2.6 to 3.5 log CFU/cm^2^ EHEC biofilm population. Lastly, displacement showed the lowest effect, by which only *P. pentosaceus* CRL 2145 significantly reduced the *E. coli* biofilm by 2.3 log CFU/cm^2^.

Furthermore, LAB cell counts remained mostly stable during growth in mixed biofilms during the three evaluated strategies. Only *L. plantarum* CRL 1482 showed a decline at 72 h when the LAB was added to pre-established EHEC biofilms (displacement). In this regard, Orihuel et al. (2018) [32] reported that EHEC reduced the growth rate of *Enterococcus mundtii* CRL 35 during planktonic co-culture; however, LAB maintained its cell counts throughout incubation, while *E. coli* entered the death phase prematurely.

In the present study, live/dead assays showed mostly EHEC cells dead within mixed biofilms containing LAB, thus corroborating the reduced EHEC counts previously observed by confocal laser scanning microscopy (CLSM) [20]. In addition, EHEC cells were surrounded by LAB in mixed biofilms, confirming the spatial localization patterns also reported by Cisneros et al. 2025 [20] through scanning electron microscopy (SEM) and CLSM. These results corroborate that, during mixed biofilm interaction, *P. pentosaceus* CRL 2145 establishes effective physical contact with *E. coli* cells, leading to disruption of their structural integrity and, consequently, cell death [20]. Jara et al. (2020) [34] showed that *Limosilactobacillus fermentum* MP26 and *Ligilactobacillus salivarius* MP14 formed microcolonies that encapsulated *Listeria monocytogenes* cells in glass-grown biofilms. This phenomenon may immobilize the pathogen, thereby enhancing the antagonistic activity of LAB.

### 4.2. Proteomic Analysis

Based on the previously established ability of *P. pentosaceus* CRL 2145 to form biofilms and hinder the attachment of *E. coli* NCTC 12900 on SS surfaces, this study investigated the differential proteomic profiles obtained during growth of the LAB strain in single- and mixed-species biofilms. This technology allowed us to explore the molecular mechanisms of LAB–EHEC interactions under sessile conditions, and how these interactions relate to physiological changes occurring during bacterial growth on SS surfaces. Comparative proteomic analysis of *P. pentosaceus* CRL 2145 in mixed biofilm (LAB–EHEC) versus single-species biofilm revealed mainly a significant overexpression with only a small number of proteins downregulated after incubation at 12 °C. This provides evidence that *P. pentosaceus* undergoes active metabolic adaptation in response to the presence of *E. coli*. The viability of *P. pentosaceus* remained nearly unchanged at 18 h, whereas the sessile population of *E. coli* was reduced by approximately 1 log unit in the mixed biofilm compared to the control, suggesting an early decline in *E. coli* growth, as observed earlier [20]. This would be advantageous for *P. pentosaceus*, which maintained stable cell numbers up to 48 h, as shown herein. This observation is consistent with the overexpression of proteins such as FtsZ and EzrA, which are involved in bacterial cell division and may facilitate dominance of LAB over *E. coli*. Additionally, the upregulation of ATP synthase α and δ subunits suggests elevated ATP production, a critical requirement for bacterial growth. Notably, stress-related proteins including DnaK, HslV, HslO, GrpE, and the general stress response regulator Gls24 were also upregulated. Preceding studies have established a correlation between the overexpression of stress proteins and the sessile lifestyle of many bacteria [35,36]. These proteins may enhance the resilience and survival of sessile *P. pentosaceus* cells under environmental stress conditions, such as 12 °C, acidic pH, and microbial competition after 18 h of incubation. The overexpression of several proteins associated with amino acid, nucleotide, carbohydrate, and lipid metabolism, as well as protein translation and post-translational modifications, indicates a metabolically active state that supports LAB persistence in the mixed biofilm. Specifically, nine overexpressed proteins related to glycolysis and the pentose phosphate pathway were identified. Phosphoenolpyruvate (PEP)-dependent sugar phosphotransferase systems may enhance glucose uptake from the meat-based medium (MES). The enzyme 6-phosphogluconate dehydrogenase, which catalyzes the production of ribulose-5-phosphate and generates NADPH, contributes to redox balance within the cell. Upregulation of phosphoglyceromutase, lactate dehydrogenase, and pyruvate oxidase further supports increased production of lactic and acetic acids, which could both act as additional antagonistic factors against EHEC. These results align with previous findings that co-culture of *Limosilactobacillus reuteri* ZJ625 and *Ligilactobacillus salivarius* ZJ614 modulates metabolite profiles and associated pathways, evidencing cell-to-cell interactions [37]. In the mixed biofilm, *P. pentosaceus* CRL 2145 also overexpressed glycerol-3-phosphate dehydrogenase, a key enzyme in the glycerol/glycerolipid pathway responsible for synthesizing glycerol-3-phosphate from glyceraldehyde-3-phosphate—the first step in phospholipid biosynthesis. Additionally, overexpression of acyl group transport proteins and glycosyltransferases associated with membrane biosynthesis suggests active fatty acid and phospholipid production. These changes likely agree with, and support, the observed increase in cell population. Consistent with Orihuel et al. (2018) [32], our data show upregulation of enzymes such as adenylosuccinate synthase (involved in de novo purine nucleotide biosynthesis), peptidases, and aminopeptidases, indicating active proteolytic metabolism in a protein rich environment such as meat processing facilities. Furthermore, upregulation of ribosomal proteins and specific aminoacyl-tRNA synthetases (e.g., prolyl-, isoleucyl-, threonyl-, and asparaginyl-tRNA ligases) points to sustained protein synthesis. Ribosomal proteins not only support ribosome assembly and stability but have also been implicated in environmental sensing and antimicrobial activity; in fact, they have been considered as emergent antimicrobial peptides [38,39]. Proteins involved in translation, including the α and β subunits of DNA-directed RNA polymerase and elongation factor Tu (Ef-Tu), were upregulated. Beyond its role in protein synthesis, Ef-Tu has been detected on the surface of various Gram-positive and Gram-negative bacteria, where it contributes to adhesion. Notably, Ef-Tu mediates *Lactobacillus reuteri* binding to sulphated carbohydrates on mucosal surfaces [40]. In addition, Ef-Tu has been reported to promote the onset of periodontitis through mediating bacteria adhesion [41]. These results point to a potential role of ribosomal proteins in EHEC inactivation during mixed biofilm development and implicate Ef-Tu in the adhesion of *P. pentosaceus* CRL 2145 to stainless steel or in enhancing its competitive advantage within the biofilm. Conversely, the pronounced repression of essential proteins in *E. coli* NCTC 12900 during co-culture biofilm formation with this LAB strain indicates that the pathogen was notably impaired in its ability to persist on stainless steel (SS) surfaces [20].

Only six proteins of *P. pentosaceus* CRL2145 were downregulated in the mixed biofilm, including SecA1 ATPase, which showed the most important under expression (FC: −528.00). As part of an ATP-dependent protein translocation system, SecA1 downregulation may indicate an energy-saving response, redirecting ATP to essential metabolic functions under competitive conditions. In contrast, other export systems like YajC were upregulated.

## 5. Conclusions

This study evidences the potentiality of selected LAB as effective biocontrol agents against *Escherichia coli* NCTC 12900 in planktonic and biofilm within a meat-based model of growth. The versatility of *P. pentosaceus* CRL 2145 is highlighted. The significant inhibitory action of culture supernatants against EHEC biofilm indicates the presence of soluble antimicrobial compounds that contribute to LAB’s antagonistic effects which are partly confirmed by the upregulation of some ribosomal proteins known as emerging antimicrobial peptides. In addition, damage and death of EHEC cells can be explained by the spatial arrangement showing LAB surrounding and possibly immobilizing EHEC cells—supporting physical and biochemical antagonistic mechanisms carried out by LAB. In summary, the combined physiological and proteomic analyses provide evidence that competition, as the most efficient inhibition strategy, involves the metabolic supremacy of the LAB strain, physical interaction, and secretion of antagonistic compounds. Proteomic analysis of *P. pentosaceus* CRL 2145 revealed a broad upregulation of proteins involved in carbohydrate metabolism, energy production, stress response, and cell division under mixed-biofilm conditions. This pattern reflects a phenotypically plastic and active adaptation in response to the stress triggered by exposure to EHEC. The upregulation of elongation factor Tu (Ef-Tu) suggests their involvement not only in enhanced protein synthesis but also in adhesion, contributing to LAB’s competitive advantage. This study provides new insights into the molecular and physiological basis of LAB–EHEC interactions and highlights *P. pentosaceus* CRL 2145 as a promising biocontrol agent for enhancing food safety in the meat industry.

## Figures and Tables

**Table 1:** Viable cell counts of lactic acid bacteria strains (log CFU/cm^2^) in single and mixed biofilms with *E. coli* NCTC 12900 under different interaction strategies (competition, exclusion, and displacement).

Batches	Bacterial Count (logCFU/cm^2^)
Competition	
*L. plantarum* CRL 1482	6.755 ± 0.784 ^ab^
*L. plantarum* CRL 1482 + *E. coli*	7.141 ± 0.342 ^a^
*L. plantarum* CRL 1075	6.515 ± 0.802 ^ab^
*L. plantarum* CRL 1075 + *E. coli*	7.522 ± 0.515 ^a^
*P. pentocaceus* CRL 2145	5.518 ± 0.502 ^bc^
*P. pentocaceus* CRL 2145 + *E. coli*	4.940 ± 0.550 ^c^
Exclusion	
*L. plantarum* CRL 1482	7.795 ± 0.535 ^a^
*L. plantarum* CRL 1482 + *E. coli*	8.360 ± 0.242 ^a^
*L. plantarum* CRL 1075	7.144 ± 0.294 ^ab^
*L. plantarum* CRL 1075 + *E. coli*	7.357 ± 1.100 ^ab^
*P. pentocaceus* CRL 2145	5.944 ± 0.138 ^b^
*P. pentocaceus* CRL 2145 + *E. coli*	6.755 ± 1.280 ^ab^
Displacement	
*L. plantarum* CRL 1482	7.045 ± 0.634 ^a^
*L. plantarum* CRL 1482 + *E. coli*	4.940 ± 0.402 ^c^
*L. plantarum* CRL 1075	6.287 ± 0.792 ^ab^
*L. plantarum* CRL 1075 + *E. coli*	5.308 ± 0.557 ^bc^
*P. pentocaceus* CRL 2145	5.118 ± 0.438 ^bc^
*P. pentocaceus* CRL 2145 + *E. coli*	4.631 ± 0.313 ^c^

Values are mean ± SD of four independent experiments (n = 4). One-way ANOVA with Tukey’s post hoc test was used; different lowercase superscripts denote significant differences between strains (*p* < 0.05).

## Data Availability

The mass spectrometry proteomics data have been deposited to the ProteomeXchange Consortium via the PRIDE partner repository [27] with the dataset identifier PXD067300.

## Supplementary Materials

The following supporting information can be downloaded at https://www.mdpi.com/article/10.3390/foods14213670/s1, Table S1: Differentially expressed proteins in sessile *Pediococcus pentosaceus* CRL 2145 cells during biofilm growth in the presence of *Escherichia coli* NCTC 12900, compared to single-species biofilm growth. Figure S1: Schematic description of the procedures for sample preparation for proteomic analysis to evaluate differential protein expression of *P. pentosaceus* CRL 2145 growth in presence or in absence of *E. coli* NCTC 12900 under biofilm conditions at 12 °C on stainless steel surface. Figure S2: *E. coli* NCTC 12900 sessile cells (log CFU/cm^2^) after the simultaneous supernatant addition on SS chips and analyzed after 48 h of incubation at 12 °C. Supernatants from 48 h LAB cultures or mixed (LAB+EHEC) co-cultures; Control (Ec control): EHEC sessile cells (log CFU/cm^2^) with addition of Meat Experimental System (MES). Assayed LAB strains: *L. plantarum* (Lp) Lp CRL 1075, Lp CRL 1482 and *P. pentosaceus* (Pp) CRL 2145 and mixed cultures (LAB+EHEC). Different letters indicate statistically significant differences (*p* < 0.05).

## Abbreviations

The following abbreviations are used in this manuscript:

MES	Meat Experimental System
LAB	Lactic Acid Bacteria
EHEC	Enterohemorrhagic *Escherichia coli*
SS	Stainless steel
