# Supplementary material for: Foodborne Lactic Acid Bacteria Inactivate Planktonic and Sessile Escherichia coli O157:H7 in a Meat Processing Environment: A Physiological and Proteomic Study
Source: Foods. 2025 Oct 28;14(21):3670. doi: 10.3390/foods14213670 (PMC12607491; doi:10.3390/foods14213670)

Supplementary material

Figure S1. Schematic description of the procedures for sample preparation for proteomic analysis to evaluate differential protein expression of P. pentosaceus CRL 2145 growth in presence or in absence of *E. coli* NCTC 12900 under biofilm conditions at 12°C on stainless steel surface

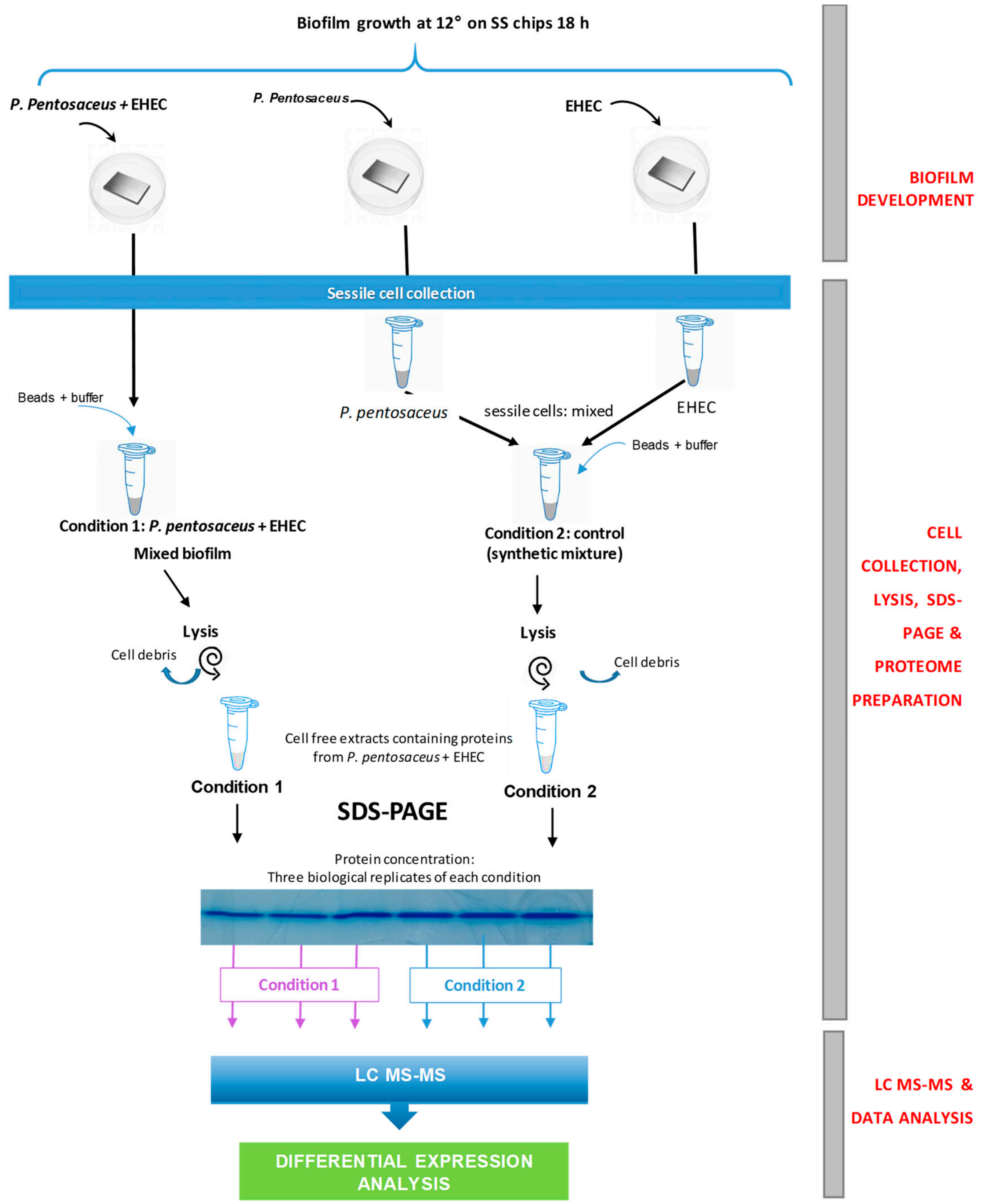

Supplement: Supplementary file 1 [file foods-14-03670-s001.zip › Supplementary material Figure S1.pdf]
