# Supplementary material for: Foodborne Lactic Acid Bacteria Inactivate Planktonic and Sessile Escherichia coli O157:H7 in a Meat Processing Environment: A Physiological and Proteomic Study
Source: Foods. 2025 Oct 28;14(21):3670. doi: 10.3390/foods14213670 (PMC12607491; doi:10.3390/foods14213670)

Figure S2. *E. coli* NCTC 12900 sessile cells (log CFU/cm^2) after the simultaneous supernatant addition on SS chips and analyzed after 48 h of incubation at 12 °C. Supernatants from 48 h LAB cultures or mixed (LAB+EHEC) co-cultures; Control (Ec control): EHEC sessile cells (log CFU/cm^2) with addition of Meat Experimental System (MES). Assayed LAB strains: *L. plantarum* (Lp) Lp CRL 1075, Lp CRL 1482 and *P. pentosaceus* (Pp) CRL 2145 and mixed cultures (LAB+EHEC). Different letters indicate statistically significant differences ($p < 0.05$).

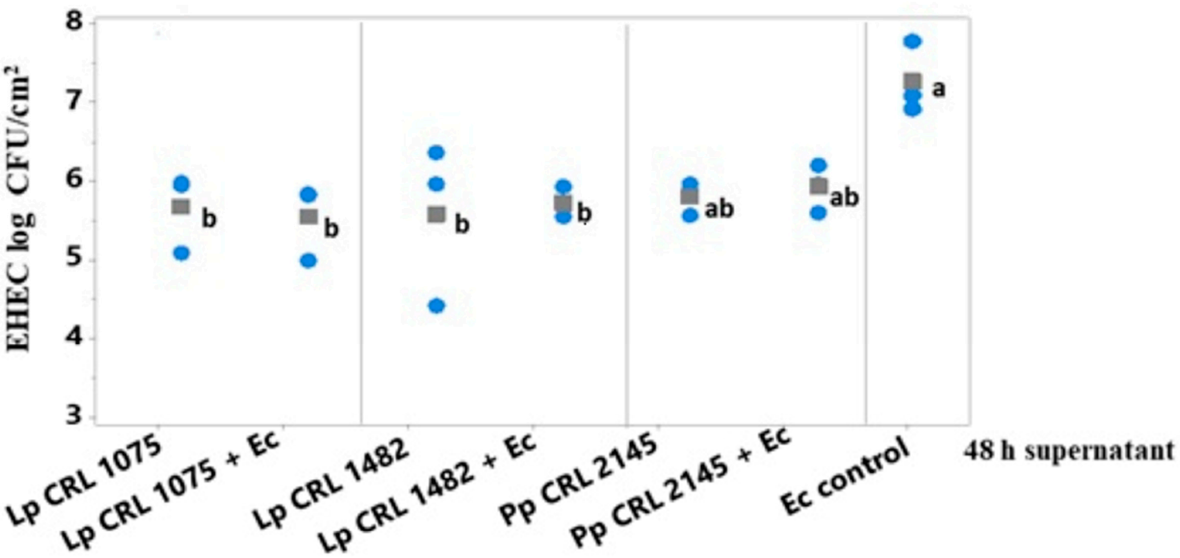

Supplement: Supplementary file 1 [file foods-14-03670-s001.zip › Supplementary material Figure S2.pdf]
