# Supplementary material for: Foodborne Lactic Acid Bacteria Inactivate Planktonic and Sessile Escherichia coli O157:H7 in a Meat Processing Environment: A Physiological and Proteomic Study
Source: Foods. 2025 Oct 28;14(21):3670. doi: 10.3390/foods14213670 (PMC12607491; doi:10.3390/foods14213670)
Supplement: Supplementary file 1 [file foods-14-03670-s001.zip › Tabla S1.pdf]

Table S1. Differentially expressed proteins in sessile *Pediococcus pentosaceus* CRL 2145 cells during biofilm growth in the presence of *Escherichia coli* NCTC 12900, compared to single-species biofilm growth.

Functional category (COG)	Accession number	gene	Protein name	Fold change (FC)	Description
**Energy production**	Q03EL2	*atpA*	ATP synthase alpha subunit	2.2	ATP synthase regulatory subunit
	Q03HR2	PEPE_0153	Pyruvate/2-oxoglutarate dehydrogenase complex, dihydrolipoamide dehydrogenase component (E3)	3.6	Glutathione reductase
	Q03EL4	*atpD*	ATP synthase, beta subunit	2.5	Part of the catalytic sites of ATP synthase
	Q03FL0	PEPE_0956	Pyrophosphate phosphohydrolase	3.2	Inorganic pyrophosphatase
	Q03EL3	*atpG*	ATP synthase gamma chain	2.6	The gamma chain is important in regulating ATPase activity and proton flow through the complex.
	Q03DT2	PEPE_1619	Uncharacterized NAD-dependent dehydrogenase (FAD)	8.5	NADH oxidase
	Q03H48	PEPE_0378	Aldo/keto reductase of the diketogulonate reductase family	3.7	Aldo keto reductase

	Q03DT1	PEPE_1620	Enzyme related to aryl-alcohol dehydrogenase	4.6	Aldo keto reductase
	Q03DT4	PEPE_1617	Malolactic enzyme	13.5	Malic enzyme
	Q03H89	PEPE_0337	Phosphate acetyltransferase	6.9	Phosphate acetyltransferase
	Q03HI1	PEPE_0244	Pyruvate/2-oxoglutarate dehydrogenase complex, component dihydrolipoamide dehydrogenase (E3), related enzyme	6.8	Glutathione reductase
	Q03HA8	PEPE_0318	Succinate dehydrogenase/fumarate reductase subunit	8.4	HI0933-like protein
	Q03EL1	*atpH*	Delta subunit of ATP synthase	3.1	F(1)F(0) ATP synthase produces ATP from ADP in the presence of a proton or sodium gradient
	Q03GA9	PEPE_0702	NADPH: Zn-dependent quinone reductase-related oxidoreductase	8.3	GroES-like domain of alcohol dehydrogenase
**Cell cycle control**	Q03F26	*tig*	Triggering factor	3.2	Participates in protein export. Acts as a chaperone, maintaining the newly synthesized protein in an open conformation. Functions as a peptidyl-prolyl cis-trans isomerase.

	Q03EY5	*ftsZ*	Cell division protein FtsZ	5.3	Essential cell division protein that forms a contractile ring structure (Z ring) at the site of future cell division. Binds GTP and exhibits GTPase activity.
	Q03EN6	*ezrA*	Septation ring formation regulator EzrA	2.8	Modulates the frequency and position of FtsZ ring formation. Inhibits FtsZ ring formation at polar sites.
**Amino acid metabolism**	Q03H30	PEPE_0396	ABC oligopeptide transport system, periplasmic component	3.0	ABC transporter, substrate binding protein
	Q03EK4	*glyA*	Serine hydroxymethyltransferase	2.5	Catalyzes the reversible interconversion of serine and glycine with tetrahydrofolate (THF). It also exhibits THF-independent aldolase activity toward beta-hydroxyamino acids, producing glycine and aldehydes, via a retro-aldol mechanism.
	Q03H09	PEPE_0417	Aminopeptidase	6.0	Aminopeptidase
	Q03HI7	PEPE_0234	Peptidase V, Metallo peptidase. MEROPS M20A family	3.9	Dipeptidase PepV
	Q03E34	PEPE_1507	Aminopeptidase	9.9	Peptidase C1 type family
	Q03DP5	*pepT*	Peptidase T	8.8	Cleaves the N-terminal amino acid of tripeptides
	Q03HY5	PEPE_0076	Zn-dependent dehydrogenase related to threonine dehydrogenase	2.8	Alcohol dehydrogenase
	Q03HP5	PEPE_0170	Dipeptidase	4.0	Dipeptidase

Q03HH8	PEPE_0247	Aminotransferase	7.5	Aminotransferase
Q03DF4	PEPE_1747	Dipeptidase	11.4	Dipeptidase
Q03E86	PEPE_1455	Dipeptidase	16.7	Dipeptidase
Q03H46	*pepX*	Xaa-Pro dipeptidyl peptidase	5.5	Removes N-terminal dipeptides sequentially from polypeptides having unsubstituted N-termini as long as the penultimate residue is proline
Q03E13	*proC*	Pyrroline-5-carboxylate reductase	7.6	Catalyzes the reduction of 1-pyrroline-5-carboxylate (PCA) to L-proline
Q03EZ4	PEPE_1174	Cysteine desulfurase	5.0	Class V aminotransferase
Q03GF1	PEPE_0659	Asparagine synthase (glutamine hydrolyzing)	2.6	Asparagine synthase
Q03GI9	PEPE_0596	PepS aminopeptidase, Metallo peptidase, MEROPS family M29	7.3	Thermophilic metalloprotease (M29)
Q03H47	PEPE_0379	Proline iminopeptidase	29.9	Releases N-terminal proline from various substrates
Q03DS2	*arcA*	Arginine deiminase	11.8	Catalytic activity of arginine
Q03ET0	PEPE_1251	Xaa-Pro aminopeptidase, Metallo peptidase, MEROPS M24B family	8.0	N-terminal domain of creatinase/prolidase
Q03HV4	PEPE_0111	Probable succinyl-diaminopimelate desuccinylase	13.9	succinyl-diaminopimelate desuccinylase

|

	Q03H36	PEPE_0390	Substrate-binding protein of the ABC amino acid transporter, PAAT family	4.7	ABC transporter substrate binding protein
**Nucleotide metabolism**	Q03GW7	*pgk*	Phosphoglycerate kinase	4.3	Belongs to the family of phosphoglycerate kinases
	Q03H90	*ackA*	Acetate kinase	2.3	Catalyzes the formation of acetyl phosphate from acetate and ATP. It can also catalyze the reverse reaction.
	Q03HD0	*prs*	Ribose-phosphate pyrophosphokinase SV=1	2.4	Participates in the biosynthesis of the central metabolite phospho-alpha-D-ribosyl-1-pyrophosphate (PRPP) through the transfer of the pyrophosphoryl group from ATP to the 1-hydroxyl group of ribose-5-phosphate (Rib-5-P)
	Q03DI2	PEPE_1719	Inosine-5'-monophosphate dehydrogenase	3.0	Catalyzes the NADPH-dependent irreversible deamination of GMP to IMP
	Q03ET4	*purA*	Adenylosuccinate synthetase	2.7	Plays an important role in the *de novo* pathway of purine nucleotide biosynthesis. It catalyzes the first committed step in AMP biosynthesis from IMP.
	Q03H14	*guaA*	GMP synthase [glutamine-hydrolyzing]	8.5	Catalyzes the synthesis of GMP from XMP

|

Q03EG0	*xpt*	Xanthine phosphoribosyltransferase	2.8	Converts the preformed base xanthine, a product of nucleic acid breakdown, to xanthosine 5'-mono-phosphate (XMP), so it can be reused for RNA or DNA synthesis
Q03DY0	*pyrG*	CTP synthase	17.4	Catalyzes the ATP-dependent amination of UTP to CTP using L-glutamine or ammonia as a nitrogen source. Regulates intracellular CTP levels through interactions with the four ribonucleotide triphosphates.
Q03FM3	*pyrR*	Bifunctional protein PyrR	2.7	Shows weak uracil phosphoribosyltransferase activity which is not physiologically significant.
Q03ER2	*tgt*	Queuine tRNA-ribosyltransferase	3.4	Catalyzes the base exchange of a guanine (G) residue with the tail precursor 7-aminomethyl-7-deazaguanine (PreQ1) at position 34 (anticodon wobble position) in tRNAs with GU(N) anticodons (tRNAs-Asp, -Asn, -His, and -Tyr)
Q03EG1	PEPE_1373	5-(Carboxyamino)imidazole ribonucleotide synthase	2.4	Participates in *de novo* purine biosynthesis
Q03E18	*nadE*	NH3-dependent NAD(+) synthase	3.2	Catalyzes the ATP-dependent amidation of deamido-NAD to form NAD. It uses ammonia as a nitrogen source.
Q03FT4	*pyrH*	Uridylate kinase	5.9	Catalyzes the reversible phosphorylation of UMP to UDP
Q03DQ7	PEPE_1644	Nucleoside diphosphate kinase	5.9	Belongs to the NDK family

	Q03F87	*cmk*	Cytidylate kinase	7.4	Belongs to the cytidylate kinase family. Subfamily type 1
	Q03F92	PEPE_1074	Purine deoxyribosyltransferase	-2.3	nucleoside 2-deoxyribosyltransferase
**Carbohydrate metabolism**	Q03GW5	*eno*	Enolase	4.0	Catalyzes the reversible conversion of 2-phosphoglycerate to phosphoenolpyruvate. Glycolytic pathway
	Q03F75	PEPE_1092	Pyruvate kinase	4.2	Belongs to the pyruvate kinase family.
	Q03E14	PEPE_1528	N-acetylglucosamine 6-phosphate diacetylase	2.3	Belongs to the superfamily of metallodependent hydrolases. NagA family.
	Q03EI1	PEPE_1352	Fructose bisphosphate aldolase 1	5.0	Fructose-1,6-bisphosphate aldolase, class II
	Q03H73	PEPE_0353	Probable phosphochelatase	6.2	Phosphoketolase
	Q03DJ1	*gpmA*	2,3-Bisphosphoglycerate-dependent phosphoglycerate mutase	5.5	Catalyzes the interconversion of 2-phosphoglycerate and 3-phosphoglycerate
	Q03GW6	*tpiA*	Triosephosphate isomerase	11.8	Participates in gluconeogenesis; Stereospecifically catalyzes the conversion of dihydroxyacetone phosphate (DHAP) to D-glyceraldehyde-3-phosphate (G3P).
	Q03EJ7	PEPE_1334	Mannose-6-phosphate isomerase	2.5	Catalytic activity. Converts D-mannose 6-phosphate to D-fructose 6-phosphate.
	Q03EI3	*pgi*	Glucose-6-phosphate isomerase	17.4	Belongs to the GPI family

|

Q03GY0	PEPE_0446	Alpha-phosphoglucomutase	4.0	Phosphoglucomutase phosphomannomutase, alpha-domain
Q03EX0	PEPE_1198	6-phosphogluconolactonase	8.6	Lactonase, 7-bladed beta hélix
Q03GH5	PEPE_0610	Phosphoenolpyruvate-protein phosphotransferase	5.2	General (non-sugar-specific) component of the phosphoenolpyruvate-dependent sugar phospho-transferase (sugar PTS) system.
Q03ER4	*zwf*	Glucose-6-phosphate 1-dehydrogenase	16.9	Catalyzes the oxidation of glucose 6-phosphate to 6-phosphogluconolactone
Q03H91	*nagB*	Glucosamine-6-phosphate deaminase	2.9	Catalyzes the reversible isomerization-deamination of glucosamine 6-phosphate (GlcN6P) to form fructose 6-phosphate (Fru6P) and ammonium ion
Q03EV4	PEPE_1227	Acid sugar phosphatase	11.8	Catalyzes the dephosphorylation of 2-6 carbon sugar acids in vitro
Q03DJ3	PEPE_1708	Predicted nucleoside diphosphate sugar epimerase	5.9	
Q03G98	PEPE_0713	6-phosphogluconate dehydrogenase, decarboxylating	8.3	Catalyzes the oxidative decarboxylation of 6-phosphogluconate to ribulose 5-phosphate and CO(2), with concomitant reduction of NADP to NADPH
Q03HY8	PEPE_0073	Glucose 1-dehydrogenase	10.2	Reductase
Q03F74	*pfkA*	ATP-dependent 6-phosphofructokinase	6.5	Catalyzes the phosphorylation of D-fructose 6-phosphate to fructose 1,6-bisphosphate by ATP, the first committed step of glycolysis

|

	Q03GH0	PEPE_0639	Pyruvate oxidase	12.6	Belongs to the TPP enzyme family
	Q03F00	*prs*	Putative ribose-phosphate pyrophosphokinase	4.4	Participates in the biosynthesis of the central metabolite phospho-alpha-D-ribosyl-1-pyrophosphate (PRPP) through the transfer of the pyrophosphoryl group from ATP to the 1-hydroxyl group of ribose-5-phosphate (Rib-5-P)
	Q03FT7	PEPE_0876	D-lactate dehydrogenase. LdhA	11.6	Belongs to the family of D-isomer-specific 2-hydroxyacid dehydrogenases
**Coenzyme metabolism and transport**	Q03DK2	*rbsK*	Ribokinase	5.5	Catalyzes the phosphorylation of ribose at O-5 in a reaction that requires ATP and magnesium.
	Q03GA4	PEPE_0707	NAD hydrolase metabolism of the HD superfamily	8.4	Hydrolase of the HD family
**Lipid Metabolism**	Q03FV4	PEPE_0859	3-oxoacyl-[acyl carrier protein] synthase 2	8.1	Catalyzes the condensation reaction of fatty acid synthesis by addition to a two-carbon acyl acceptor of malonyl-ACP
	Q03FU8	PEPE_0865	Enoyl-[acyl carrier protein] reductase [NADH]	3.0	Enoyl reductase [NADH]
	Q03EA1	PEPE_1440	Esterase/lipase	2.5	alpha/beta hydrolase fold
	Q03GY3	*gpsA*	Glycerol-3-phosphate dehydrogenase [NAD(P)+]	4.4	Glycerol-3-phosphate dehydrogenase

|

	Q03FV2	PEPE_0861	(3R)-hydroxymyristoyl-[acyl-carrier-protein] dehydratase	-2.1	FabA-like domain
	Q03HR9	PEPE_0146	Short-chain alcohol dehydrogenase	6.7	SD family oxidoreductase
	Q03HY8	PEPE_0073	Glucose 1-dehydrogenase	10.2	Reductase
	Q03FV7	*acpP*	Acyl group transport protein	10.3	Carrier of the growing fatty acid chain in fatty acid biosynthesis.
**Translation**	Q03EB9	*rplB*	Ribosomal protein 50S L2	2.3	Required for the formation of the 70S ribosome, for the binding of tRNA and the formation of peptide bonds. It has been suggested to have peptidyltransferase activity.
	Q03F88	PEPE_1079	Ribosomal protein SSU S1P	2.3	Ribosomal protein S1
	Q03EC8	*rplE*	Ribosomal protein 50S L5	2.9	Binds and probably mediates the binding of 5S RNA to the large ribosomal subunit. On the 70S ribosome, it contacts the S13 protein of the 30S subunit (bridge B1b), connecting the two subunits.
	Q03EB8	*rplW*	Ribosomal protein 50S L23	2.1	Assembly protein binds to 23S rRNA
	Q03ED2	*rplR*	Ribosomal protein 50S L18	3.0	Binds and probably mediates the binding of 5S RNA to the large ribosomal subunit, forming part of the central protuberance
	Q03FT6	*rpsB*	Ribosomal protein 30S S2	2.6	Belongs to the universal family of ribosomal proteins uS2

Q03EB3	*rpsG*	Ribosomal protein 30S S7	3.5	rRNA-binding protein, binds directly to 16S rRNA and nucleates the assembly of the principal domain of the 30S subunit. It probably blocks the exit of tRNA from the E site.
Q03ET7	*efp*	Elongation factor P	3.2	Participates in the synthesis of peptide bonds
Q03EB4	*fusA*	Elongation factor G	2.5	Catalyzes the coordinated movement of the two tRNA molecules, the mRNA, and conformational changes in the ribosome.
Q03I50	*rplI*	Ribosomal protein 50S L9	3.2	Binds to 23S rRNA
Q03EK1	*prfA*	Peptide chain release factor 1	2.5	Directs translation termination in response to peptide chain termination codons UAG and UAA
Q03GN7	*asnS*	Asparagine--tRNA ligase	10.6	Aminoacylt-tRNA synthetase
Q03E49	*rplA*	Ribosomal protein 50S L1	4.1	Binds directly to 23S rRNA. The L1 stem is quite mobile on the ribosome and is involved in the release of tRNA from the E site.
Q03EE9	*rpsI*	Ribosomal protein 30S S9	2.1	Belongs to the universal family of ribosomal proteins uS9
Q03EY9	*ileS*	Isoleucina--tRNA ligase	5.0	Catalyzes the binding of isoleucine to tRNA
Q03EC3	*rplP*	Ribosomal protein 50S L16	3.0	Binds to 23S rRNA and is also seen to make contact with A and possibly P site tRNAs.
Q03FS9	*proS*	Proline--tRNA ligase	5.1	Catalyzes the binding of proline to tRNA (Pro) in a two-step reaction

Q03FZ8	*efp*	Elongation factor P	2.3	Participates in the synthesis of peptide bonds.
Q03F23	*rnj*	Ribonuclease J	3.2	An RNase that has 5'-3' exonuclease and possibly endonuclease activity. Involved in rRNA maturation and, in some organisms, also in the maturation or decay of mRNA.
Q03GB7	*thrS*	Threonine-tRNA ligase	4.2	Catalyzes the binding of threonine to tRNA (Thr) in a two-step reaction
Q03ED4	*rpmD*	Ribosomal protein 50S L30	4.9	Ribosomal protein L30
Q03E48	*rplK*	Ribosomal protein 50S L11	2.9	Part of the ribosomal stem that helps the ribosome interact with GTP-bound translation factors.
Q03FT5	*tsf*	Elongation factor Ts	14.4	Associated with the EF-Tu.GDP complex and induces the exchange of GDP to GTP
Q03G83	*rpmG1*	Ribosomal protein 50S L33 1	6.5	Belongs to the family of bacterial ribosomal proteins bL33
Q03E40	*gltX*	Glutamate-tRNA ligase	8.1	Catalyzes the binding of glutamate to tRNA (Glu) in a two-step reaction
Q03EG5	*gatA*	Glutamyl-tRNA(Gln) amidotransferase subunit A	5.7	Allows the formation of correctly charged Gln-tRNA(Gln) through the transamidation of misacylated Glu-tRNA(Gln) in organisms that lack glutaminyl-tRNA synthetase.

	Q03HF9	*metG*	Methionine--tRNA ligase	3.5	Required not only for the elongation of protein synthesis, but also for the initiation of all mRNA translation through aminoacylation of the initiator tRNA (fMet)
	Q03GW3	*rnr*	Ribonuclease R	3.95	3'-5' exoribonuclease that releases 5' nucleoside monophosphates and is involved in the maturation of structured RNAs
	Q03ER8	*alaS*	Alanine--tRNA ligase	7.2	Catalyzes the binding of alanine to tRNA (Ala) in a two-step reaction
	Q03EP0	*valS*	Valine--tRNA ligase	110.9	Catalyzes the binding of alanine to tRNA (Val) in a two-step reaction
	Q03H31	*hflX*	GTPase HflX	-2.6	GTPase that associates with the 50S ribosomal subunit and may play a role during protein synthesis or ribosome biogenesis
	Q03F63	*era*	GTPase Era	-4.6	An essential GTPase that binds both GDP and GTP, with rapid nucleotide exchange. It plays a role in 16S rRNA processing and 30S ribosomal subunit biogenesis, and possibly also in the regulation of the cell cycle and energy metabolism.
**Transcription**	Q03EE2	*rpoA*	DNA-directed RNA polymerase alpha subunit DNA-directed	3.1	DNA-dependent RNA polymerase catalyzes the transcription of DNA into RNA using the four ribonucleoside triphosphates as substrates.

|

	Q03EB0	*rpoC*	DNA-directed RNA polymerase beta subunit	4.5	DNA-dependent RNA polymerase catalyzes the transcription of DNA into RNA.
	Q03ET1	PEPE_1250	Catabolite control protein A	3.2	Catabolite control protein A.
	Q03DQ3	PEPE_1648	Transcriptional regulator	3.1	Transcriptional regulator of the MarR family
	Q03FS6	*nusA*	Transcription termination/antitermination protein NusA	25.3	Participates in both transcription termination and antitermination.
	Q03I59	PEPE_0002	Beta-sliding clamp	2.6	Confers DNA anchoring and processivity to DNA polymerases and other proteins. It acts as a clamp, forming a ring around the DNA.
**Recombination, replication and repair**	Q03EQ5	recA	RecA protein	375	Catalyzes ATP hydrolysis in the presence of DNA and ATP-dependent hybridization of homologous single-stranded DNA. Interacts with LexA, causing its activation and leading to its autocatalytic cleavage.
	Q03DT8	PEPE_1613	Exodeoxiribonuclease III	9.8	Exodeoxiribonuclease III.
**Cell wall and membrane biogenesis**	Q03HK5	PEPE_0216	Lipopolysaccharide biosynthesis glycosyltransferase	4.3	Glycosyltransferase activity.
	Q03GY2	PEPE_0444	UTP-glucose-1-phosphate uridylyltransferase	6.0	UTP-glucose-1-phosphate uridylyltransferase.

	Q03GD4	*prsA*	PrsA folding protein	5.0	Plays an important role in protein secretion by assisting in extracellular folding following translocation of several secreted proteins.
	Q03GU9	*glmS*	Glutamine-fructose-6-phosphate aminotransferase [isomerizing]	9.0	Catalyzes the first step in hexosamine metabolism, converting fructose-6P to glucosamine-6P using glutamine as a nitrogen source.
	Q03GC5	*murC*	UDP-N-acetylmuramate-L-alanine ligase	-3.5	Belongs to the MurCDEF family.
**Post-translational modifications**	Q03FR7	*dnaK*	Chaperone protein DnaK	3.5	Heat shock protein 70 kDa
	Q03H05	*groEL*	Chaperonine GroEL	4.1	Prevents misfolding and promotes proper refolding and assembly of unfolded polypeptides generated under stress conditions
	Q03EN9	PEPE_1292	Thiol peroxidase (atypical 2-Cys peroxiredoxin)	2.8	Thiol-specific peroxidase that catalyzes the reduction of hydrogen peroxide and organic hydroperoxides to water and alcohols, respectively. It participates in cellular protection against oxidative stress.
	Q03FK3	*hslV*	ATP-dependent protease subunit HslV	5.7	Protease subunit of a proteasome-like degradation complex that is thought to be a general protein degradation machinery.

|
	Q03FK4	PEPE_0962	ATP-dependent protease HslVU (ClpYQ), ATPase subunit	6.7	This subunit has chaperone activity. The binding of ATP and its subsequent hydrolysis by HslU are essential for the cleavage of protein substrates subsequently hydrolyzed by HslV. HslU recognizes the N-terminal portion of its protein substrates and unfolds them before they are guided to HslV for hydrolysis.
	Q03GH8	PEPE_0607	ATP-binding subunit of Clp protease and DnaK/DnaJ chaperones	7.1	It belongs to the ClpA ClpB family
	Q03FR8	*grpE*	Protein GrpE	5.5	Actively participates in the response to hyperosmotic and thermal shock by preventing the aggregation of stress-denatured proteins, in association with DnaK and GrpE.
	Q03HC3	PEPE_0302	Neutral endopeptidase	19.0	M13 peptidase family
	Q03E07	*hslO*	33 kDa chaperonine	16.3	Redox-regulated molecular chaperone. It protects both thermally unfolded and oxidatively damaged proteins from irreversible aggregation. It plays an important role in the bacterial defense system against oxidative stress.
**Inorganic ion metabolism**	Q03FQ8	PEPE_0906	Catalase containing manganese	6.9	Catalase activity
	Q03G74	PEPE_0737	Cystathionine beta-lyase family protein	3.7	aluminum resistance
	Q03H26	PEPE_0400	ABC oligopeptide transport system, ATPase component	2.4	Belongs to the ABC transporter superfamily.

**Signal transduction**	Q03F12	*bipA*	50S ribosomal subunit assembly factor BipA	2.3	GTP-binding protein TypA
	Q03F25	*tuf*	Elongation Factor Tu	2.3	Promotes GTP-dependent binding of aminoacyl-tRNA to the A site of ribosomes during protein biosynthesis.
**Intracellular trafficking**	Q03ER3	PEPE_1268	Translocase protein subunit yajC	71.0	Preprotein translocase
	Q03GZ8	*secA1*	Translocase protein. SecA subunit 1	-528.0	Plays a central role in the transfer of proteins to and across the cell membrane, serving as an ATP-driven molecular motor that propels the stepwise translocation of polypeptide chains across the membrane.
**Defense mechanisms**	Q03I42	PEPE_0019	Putative hemin import ATP-binding protein HrtA	4.9	Part of the ABC transporter complex hrt involved in hemin import. Responsible for energy coupling to the transport system
	Q03DF8	PEPE_1743	ABC-type antimicrobial peptide transport system, ATPase component	22.8	ABC transporter, ATP-binding protein
**Unknown function**	Q03G71	PEPE_0740	Exonuclease SbcC	3.9	-
	Q03F07	*rpoY*	Epsilon subunit of DNA-directed RNA polymerase	3.4	A non-essential component of RNA polymerase (RNAP)
	Q03HJ1	PEPE_0230	DUF871 domain-containing protein	3.0	Bacterial protein of unknown function (DUF871)
	Q03E89	PEPE_1452	T30S ribosomal protein S20	8.5	unknown function

|
Q03EJ8	*gatD*	GatD subunit of lipid II isoglutaminyl synthase (glutamine hydrolyzing)	2.2	Glutamine amidotransferase
Q03H79	PEPE_0347	Predicted oxidoreductase	5.8	Aldo keto reductase
Q03DT0	PEPE_1621	Predicted hydrolase (HAD superfamily)	3.2	Haloacid dehalogenase-like hydrolase
Q03DN3	PEPE_1668	Stress response regulator gls24 homolog	4.5	Asp23/Gls24 family envelope stress response protein Bacterial protein of unknown function
Q03FZ7	PEPE_0815	Alkaline shock protein	2.5	Asp23 family, function related to the cell envelope
Q03DZ5	PEPE_1556	CBS domain-containing protein	113.0	CBS Domain
Q03DN0	PEPE_1671	Alkaline shock response membrane anchoring protein AmaP	10.5	-
Q03DW8	PEPE_1583	Predicted phosphatase	4.9	Haloacid dehalogenase-like hydrolase
Q03GD5	PEPE_0676	Predicted HD superfamily hydrolase	15.7	Metal-dependent phosphohydrolases with a conserved 'HD' motif.

Expression data (FC) result from the performed statistical analyses ($p<0.05$; FC>2) using the Proteome Discoverer and Perseus software. Functional classification of the identified proteins and detection of clusters of orthologous groups (COGs) were carried out using the Universal Protein Resource (UniProt) database and the EggNOG online framework.
